# Range-wide assessment of habitat suitability for jaguars using multiscale species distribution modelling

**DOI:** 10.1038/s41598-025-30512-5

**Published:** 2025-12-24

**Authors:** Guilherme Costa Alvarenga, Caroline C. Sartor, Samuel A. Cushman, Alexandra Zimmermann, Ana Carolina Srbek-Araujo, Ana Cristina Mendes-Oliveira, Bart Harmsen, Carlos De Angelo, Carolina Franco Esteves, Claudia B. de Campos, Daiana Jeronimo Polli, Diego F. Passos Viana, Diogo Maia Gräbin, Emiliano Donadio, Emiliano E. Ramalho, Esteban Payán, Fernando C. C. Azevedo, Francisco Palomares, George V. N. Powell, Gerardo Ceballos, Grasiela Porfirio, Heliot Zarza, Ivonne Cassaigne, Juliano A. Bogoni, Leonardo Sena, Louise Maranhão, Marcos Roberto Monteiro de Brito, Mathias W. Tobler, Øystein Wiig, Rebecca J. Foster, Ricardo Sampaio, Rodrigo Nuñez, Ronaldo G. Morato, Valeria Boron, Wener Hugo Arruda Moreno, Yadvinder Malhi, David W. Macdonald, Żaneta Kaszta

**Affiliations:** 1 Wildlife Conservation Research Unit (WildCRU), Department of Biology, University of Oxford, Life and Mind Building, South Parks Road, Oxford, OX1 3EL UK; 2 Grupo de Pesquisa em Ecologia e Conservação de Felinos na Amazônia, Mamirauá Institute for Sustainable Development (MISD), Estrada do Bexiga, nº 2584, Tefé, AM Brazil; 3 Department of Biological Sciences, Northern Arizona University, Flagstaff, AZ USA; 4 Programa de Pós-Graduação em Ciência Animal e Programa de Pós-Graduação em Ecologia de Ecossistemas, Universidade Vila Velha, Av. Comissário José Dantas de Melo, 21, Boa Vista, Vila Velha, Espírito Santo 29102-920 Brazil; 5 Laboratório de Ecologia e Zoologia de Vertebrados (LABEV/ICB), Universidade Federal do Pará, Belém, PA Brazil; 6 Panthera, 104 West 40th Street, 5th Floor, New York, NY 10018 USA; 7 Instituto de Ciencias de la Tierra, Biodiversidad y Ambiente (IBCIA), Universidad Nacional de Río Cuarto and National Scientific and Technical Research Council (CONICET), Ruta Nacional 36 Km 601, Río Cuarto, Argentina; 8 Institute for the Conservation of Neotropical Carnivores, Avenida Horácio Neto, 1030, Parque Edmundo Zanoni, Atibaia, SP Brazil; 9 Programa de Pós-Graduação em Ecologia e Conservação, Instituto de Biociências – Inbio, Universidade Federal de Mato Grosso do Sul, Campo Grande, MS 79070-900 Brazil; 10 Fundación Rewilding Argentina, Scalabrini Ortiz 3355, 1425 Buenos Aires, Argentina; 11 WCS Big Cat Program, New York, USA; 12 Departamento de Ciências Naturais, Universidade Federal de São João del Rei, São João del Rei, MG 36301-160 Brazil; 13 Department of Conservation Biology, Doñana Biological Station, CSIC, Avda. Américo Vespucio 26, 41092 Isla de la Cartuja, Seville Spain; 14 Wildlife Protection Solutions, 2501 Welton Street, Denver, CO 80205 USA; 15 Laboratorio de Ecología y Conservación de Fauna Silvestre, Instituto de Ecología, Universidad Nacional Autónoma de México, Ciudad Universitaria, Coyoacán, Ciudad de México, Mexico; 16 Instituto Homem Pantaneiro, Corumbá, Mato Grosso do Sul Brazil; 17 Departamento de Ciencias Ambientales, Universidad Autónoma Metropolitana, Unidad Lerma, CBS, Lerma de Villada, Mexico; 18 Primero Conservation, Box 158885935, Pinetop, AZ USA; 19 Centro de Pesquisa de Limnologia, Biodiversidade e Etnobiologia do Pantanal; Laboratório de Mastozoologia; Programa de Pós-graduação em Ciências Ambientais, Universidade do Estado de Mato Grosso-UNEMAT, Cáceres, MT 78217-900 Brazil; 20 Mamirauá Institute for Sustainable Development (MISD), Estrada do Bexiga, nº 2584, Tefé, AM Brazil; 21 Associação Onçafari, São Paulo, SP Brazil; 22 San Diego Zoo Wildlife Alliance, Conservation Science and Wildlife Health, 15600 San Pasqual Valley Road, Escondido, CA 92027 USA; 23 Natural History Museum, University of Oslo, POB 1172 Blindern, 0318 Oslo, Norway; 24 Centro Nacional de Pesquisa e Conservação de Mamíferos Carnívoros (CENAP-ICMBio), Estrada Municipal Hisaichi Takebayashi, 8600, Atibaia, SP 12952-011 Brazil; 25 Alianza Jaguar AC, Lab. Vida Silvestre, Fac. Biol. Universidad Michoacana, Morelia, Michoacán Mexico; 26 World Wide Fund for Nature (WWF) UK, The Living Planet Centre, Brewery Road, Woking, GU214LL UK; 27 Environmental Change Institute, School of Geography and the Environment, University of Oxford, South Parks Road, Oxford, UK; 28 Leverhulme Centre for Nature Recovery, University of Oxford, South Parks Road, Oxford, UK

**Keywords:** Habitat suitability, Indigenous lands, Jaguar conservation units, Multiscale modelling, Panthera onca, Protected areas, Ecology, Ecology, Environmental sciences

## Abstract

Jaguars (*Panthera onca*) are highly sensitive to persecution, habitat loss, and fragmentation, making the identification of suitable habitat critical for conservation planning. Using GPS telemetry data from 172 individuals across eight countries – the largest jaguar dataset to date – we developed multiscale Resource Selection Functions (RSFs) incorporating 15 environmental covariates to model habitat suitability across the species’ historic range. Jaguars selected productive habitats near water and strongly avoided human-modified landscapes, including areas with high human population density and livestock presence. The resulting habitat suitability surface showed strong predictive performance (AUC = 0.88; Boyce Index = 0.91) and correlated with known density estimates and distribution models. Jaguar Conservation Units (JCUs) and Protected Areas (PAs) contained 68.7% and 53.9% of predicted suitable habitat, respectively, while occupying only a third of the range. Non-designated lands, though comprising just 4% of the range, held nearly 10% of total suitability. The Amazon and Mayan Forests were identified as core strongholds, while ecoregion-based modelling revealed additional areas of high suitability in the Pantanal, Gran Chaco, Cerrado, and coastal Mexico. While Brazil encompassed the largest extent of highly suitable habitat, countries such as Paraguay, Argentina, and the United States gained conservation relevance under the ecoregion-stratified scenario.

**Supplementary Information:**

The online version contains supplementary material available at 10.1038/s41598-025-30512-5.

## Introduction

Large carnivores occupy the top of the food chain and consequently have naturally low population sizes, which makes them particularly susceptible to ecological perturbations^1^. For example, jaguars (*Panthera onca*) historically occurred from the southwestern United States (US) to central Argentina^2^, but their range has been reduced by half over the last century mainly due to habitat loss and persecution^3^. Within their remaining range, the Amazonian biome, the wetlands of the Pantanal and the Mayan Forest still harbour large jaguar populations, while elsewhere they persist in smaller, isolated and generally highly threatened populations^4,5^. However, considerable variation exists in jaguars’ habitat use and population densities even within these core areas, reflecting underlying differences in habitat quality and environmental pressures^6–8^. This study aims to refine our understanding of how jaguars respond to environmental and landscape changes by identifying critical habitat areas through the application of resource selection functions, remotely sensed predictors, and movement data spanning the species’ historic range.

Resource selection functions (RSFs) have been widely used to quantify, predict and map interactions between species and their environments, and are well suited to defining important habitats (and habitat elements) for the species viability^9^. RSFs compare the used habitat (usually, the presences extracted from animal locations) with the available habitat to quantify the variation in the use of resources^10^. Ideally, “used” and “available” data should be temporally synchronized, ensuring that predictions are aligned with the actual choices made by the species/animal. In addition, species perceive and respond to the landscape features in different spatial scales, therefore defining the “scale of effect” in which each landscape feature has on the target species helps to avoid erroneous outcomes^11,12^. New methods, such as scale optimization approaches^13,14^, enable precise tuning of the species-environmental relationship to scale within the context of RSF modelling. Nevertheless, applying such techniques relies heavily on having adequate empirical data, which is often challenging to acquire for elusive species like jaguars.

The first maps delineating the core habitat areas for jaguars were developed solely based on expert knowledge, as empirical data were scarce. These maps identified 130 areas (Jaguar Conservation Units – JCUs) considered crucial for the long-term survival of the species although not necessarily formally protected^15^. Recently, as the emergence of new technologies has enabled the collection of large empirical data sets on jaguar occurrence and movement, researchers have been regionally evaluating JCUs^16–19^. Those studies have used various methods, and scales, and have focused on different geographical regions; nevertheless, some general patterns in the jaguar’s response to the landscape have emerged. For instance, the importance of protected areas and Indigenous Lands (“PAs” for simplicity) for jaguar populations is notable throughout its geographical range, especially outside the Amazon biome, where all the remaining suitable habitats are fragmented^20–22^. Among landscape features and land cover types, primary and secondary forests, and water bodies, are consistently identified as the most important predictors of high habitat suitability and dispersal corridors for jaguars^23–25^. Conversely, jaguars generally avoid areas with high human densities and a high human footprint index, such as settlements, crop farms and roads^26–28^. In addition, it is well documented that male and female jaguars use their habitat differently. Compared to females, males have larger home ranges, use trails and secondary roads more intensively, and are less sensitive to human presence and activities^29–31^ - although, recent studies have highlighted variation in these patterns^32,33^. Despite growing knowledge of jaguars’ spatial requirements, empirically derived spatial data remain limited, posing a significant challenge across the species range.

To date, only two range-wide studies on jaguar distribution have been conducted, both of which relied on compiled occurrence data from multiple sources with varying levels of accuracy and quality^34,35^. Additionally, neither study explored scale optimization, temporally matched observations to predictor variables, or accounted for sex differentiation. These methodological limitations, while understandable at the time, may have contributed to misleading or inaccurate predictions^36,37^ and likely contributed to the incongruence of distribution predictions^34,35^.

Here, we leverage the largest dataset on jaguar movement ever compiled, integrating a multi-scale approach, ensuring temporal alignment of datasets where available and sex-specific behavioural variation to model habitat suitability across the species’ historical range. Using data from 172 GPS-collared jaguars, we aimed to produce the most up-to-date map of jaguar proportional probability of occurrence and assess its conservation implications. Specifically, we evaluate the effectiveness of JCUs and PAs in safeguarding critical jaguar habitats, analyse the distribution of suitable habitats across countries and, as a case study, estimate the proportion of these habitats within non-designated lands in Brazil. This effort represents a unique contribution to jaguar research and conservation, providing governments and stakeholders with essential information to guide conservation planning and policy.

## Results

### Resource selection

After cleaning erroneous data and filtering locations that fell within the same pixel, 29,962 GPS-locations were retained for model development. Females contributed 12,302 locations, and males 17,660 (Table 1). Jaguars responded to the spatial covariates at varying scales with no clear preference for a specific scale (Table 2). From the retained covariates in the final models, only roughness was not significantly associated with jaguar habitat use. Our model indicated that jaguars were highly averse to anthropogenic features with human population density, livestock and human modification presenting the three strongest negative effects on the species. Conversely, soil CEC, NDVI, and water surface had the strongest positive effect in the model, indicating jaguars are more likely to select productive habitats closer to water bodies (Table 2).

**Table 1:** Number (Nº) of Jaguars (*Panthera onca*) and GPS-locations per biome.

Biome	Nº locations (Individuals)		
	Females	Males	Total
Amazon	1350 (10)	3565 (16)	6145 (26)
Atlantic Forest	1686 (14)	2445 (10)	5164 (24)
Caatinga	958 (2)	2038 (4)	3746 (6)
Cerrado	411 (2)	1253 (4)	2079 (6)
Dry Chaco	354 (1)	2451 (6)	3505 (7)
Thornscrub of Sonora (Mexico)	445 (1)	511 (1)	1195 (2)
Humid Chaco	1626 (10)	572 (3)	2746 (13)
Llanos	22 (1)	64 (1)	108 (2)
Mangroves	78 (2)	257 (5)	418 (7)
Pantanal	3318 (31)	2867 (26)	7731 (57)
Tropical Forests of Central America	2054 (12)	1637 (10)	4616 (22)
Total	12,302 (86)	17,660 (86)	29,962 (172)

**Table 2:** Multi-scale generalized linear mixed model (GLMM) predicting Jaguar (*Panthera onca*) habitat suitability across the species historic range.

Covariates	Scale (km)	β coefficient	Std. Error	Pr (>|z|)
Temperature seasonality	16	−0.268	0.050	< 0.001
Surface temperature	1	−0.232	0.010	< 0.001
Human modification	4	−0.484	0.018	< 0.001
Human population density	8	−5.713	0.186	< 0.001
Livestock	8	−1.041	0.020	< 0.001
Compound topographic Index	2	0.193	0.014	< 0.001
Elevation	0.5	−0.271	0.015	< 0.001
Roughness	4	−0.001	0.016	0.942
Topographic position Index	32	−0.210	0.008	< 0.001
Cation exchange capacity	4	0.525	0.018	< 0.001
Water surface	16	0.235	0.007	< 0.001
Forest degradation	2	−0.042	0.010	< 0.001
Normalized Difference Vegetation Index	0.5	0.300	0.010	< 0.001
Defaunation Index	32	−0.241	0.015	< 0.001
Burned areas	1	−0.061	0.007	< 0.001

Std. Error, adjusted standard error; Pr (>|z|), significance.

### Model prediction

**Fig. 1:**
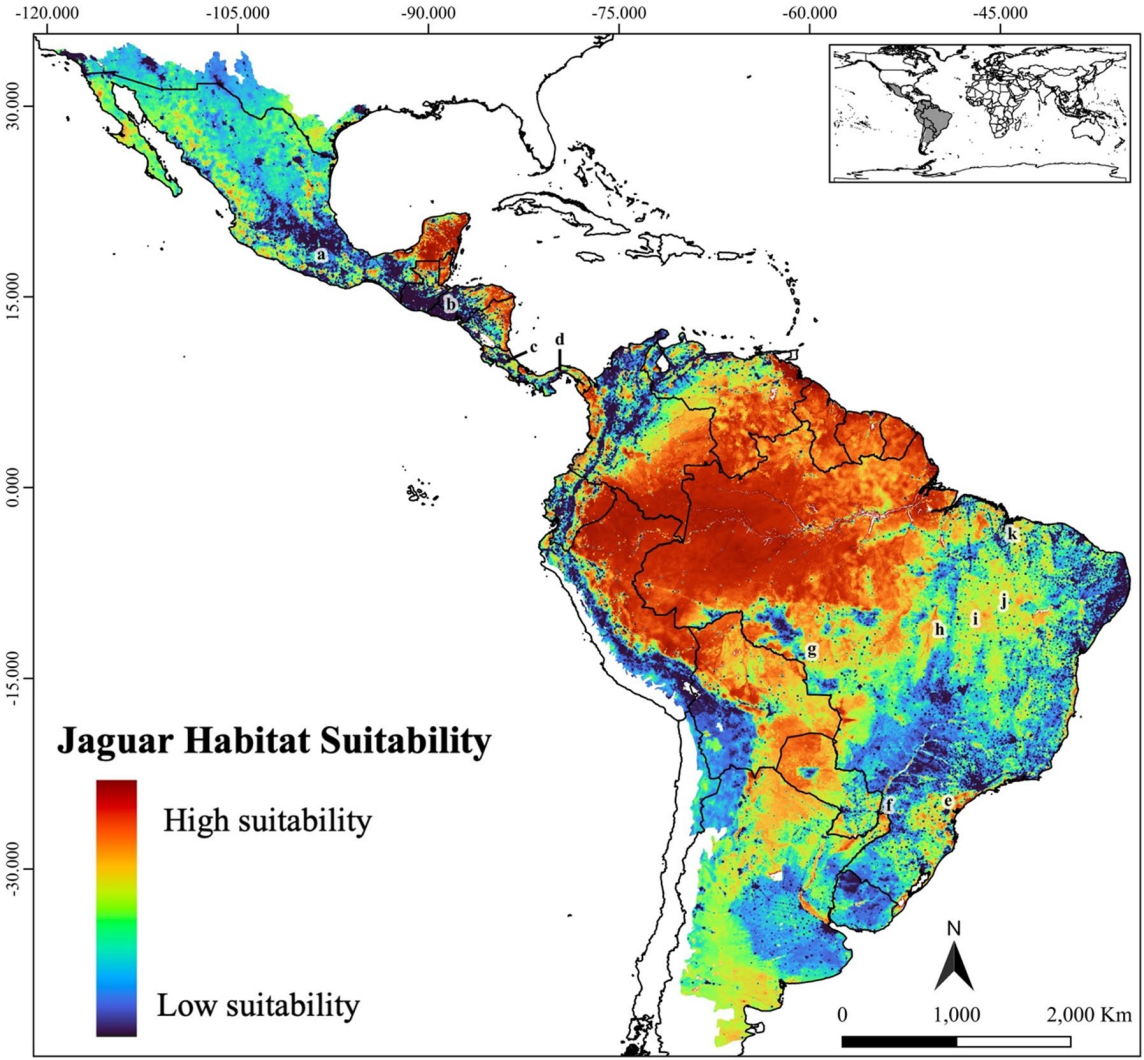
Predicted habitat suitability for jaguars (*Panthera onca*) across the species historical range. Letters mark specific regions of interest discussed in detail in the Discussion section. Figure created using QGIS v3.36.0 (https://qgis.org).

Using the linear combination of variables in our optimized model, we predicted the habitat suitability surface for jaguars across their historical range (Fig. 1). Forested environments were the most suitable habitats for the species. The Amazon biome and the Mayan forests in southeast Mexico represented the two largest continuous areas of predicted suitable habitat. Additionally, the Gran Chaco from Paraguay and northern Argentina, the tropical forests across Central America, the wetlands of Pantanal on the borders between Brazil, Paraguay and Bolivia, and the remaining patches of Atlantic Forest were also predicted to have relatively high suitability. Large areas of intermediate suitability were found from northwest Mexico in Sonora through the west coast until the Sinaloa region, but also in the Oaxaca region in southern Mexico, as well as in the Cerrado and part of the Caatinga biome. Interestingly, the predictions identified as intermediate suitability some peripheral regions where jaguar no longer present – or may have never occurred – such as Baja California (Mexico), the gulf coastal plains in southern Texas (US) and central and southern Argentina in the extreme south of the species potential historical distribution (Fig. 1).

The two validation tests indicated our suitability surface was highly predictive: Boyce Index (*f* = 0.91; Fig. S2) and AUC = 0.88 (sensitivity = 0.93, specificity = 0.77). Additionally, the jaguar density estimates were positively related to our suitability surface (β = 4.42, SE = 1.20, t = 3.7, *p* < 0.0001, 95% CI = [2.03, 6.82], *R*^2^ = 0.14) (Supplementary Fig. S3), and the correlation test demonstrated our prediction map is highly correlated with the distribution model by Jędrzejewski et al.^34^ (*r* = 0.72).

### Conservation implications

Considering the total summed habitat suitability across the jaguar’s current range – where each pixel contributes to the total according to its suitability value – non-designated lands account for 9.83% of the total, despite occupying only 4.0% of the range. Most of these lands are located in the central-western Brazilian Amazon. In comparison, JCUs and PAs concentrate 68.7% and 53.9% of the predicted habitat suitability while covering just 33.0% and 29.3% of the range, respectively (Supplementary Table S3).

**Fig. 2:**
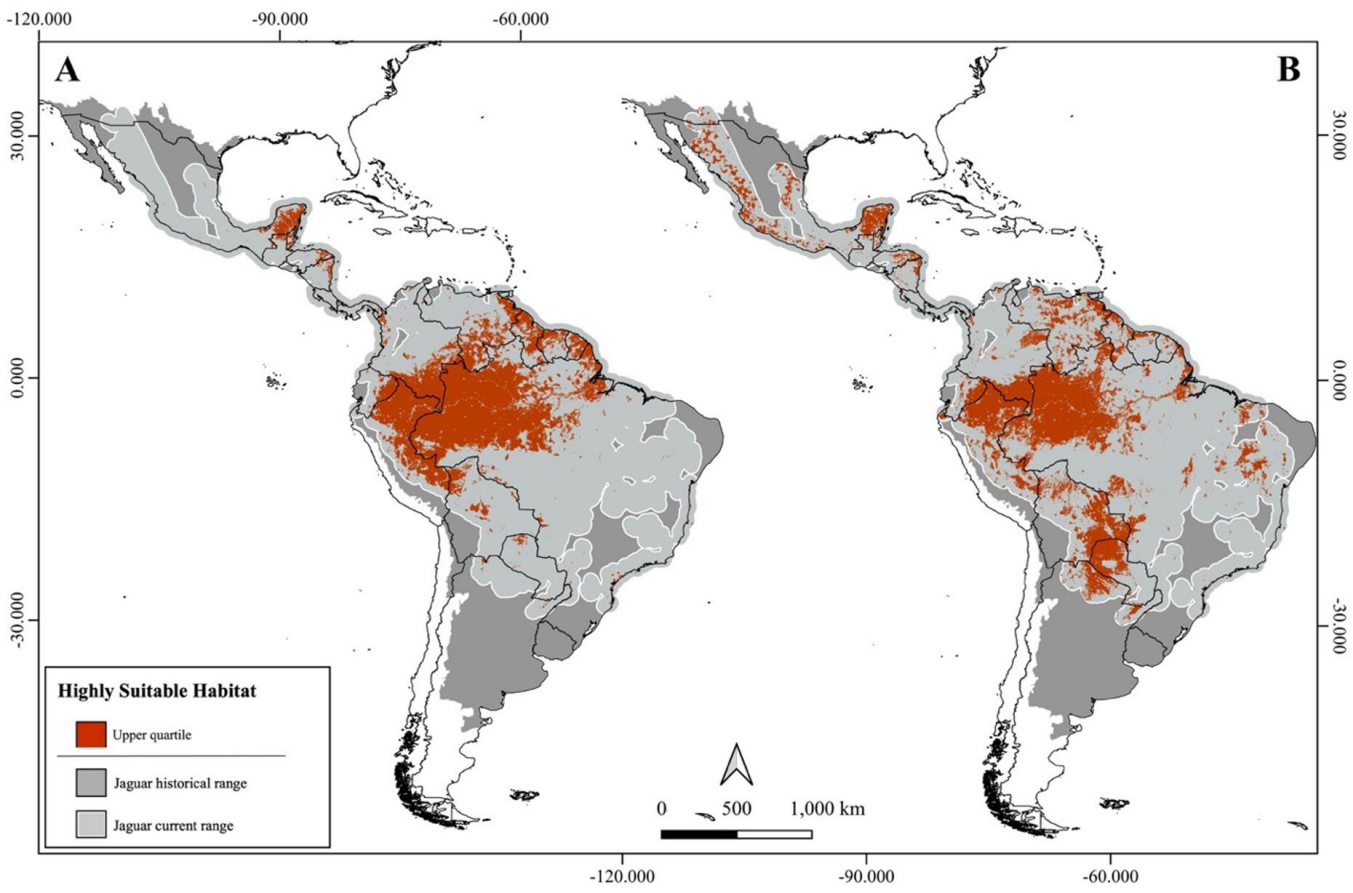
The jaguar highly suitable habitats considering the range-based (**A**) and ecoregion-based scenarios (**B**). Figure created using QGIS v3.36.0 (https://qgis.org).

**Fig. 3:**
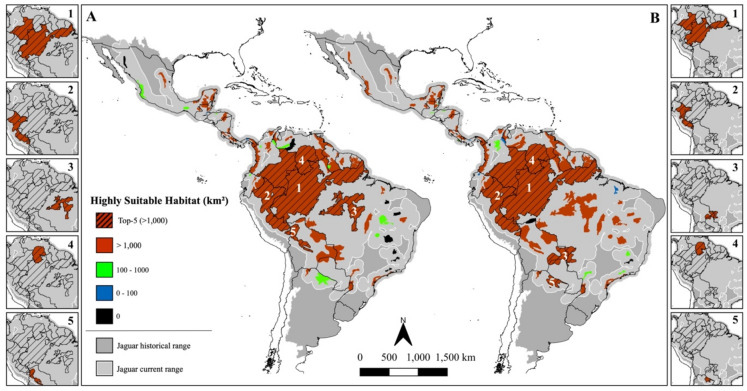
The Jaguar Conservation Units (JCUs) classified by the amount of highly suitable area for jaguars considering the range-based (**A**) and ecoregion-based scenarios (**B**). The hashed pattern and side maps underline the five main JCUs in each scenario. Figure created using QGIS v3.36.0 (https://qgis.org).

**Fig. 4:**
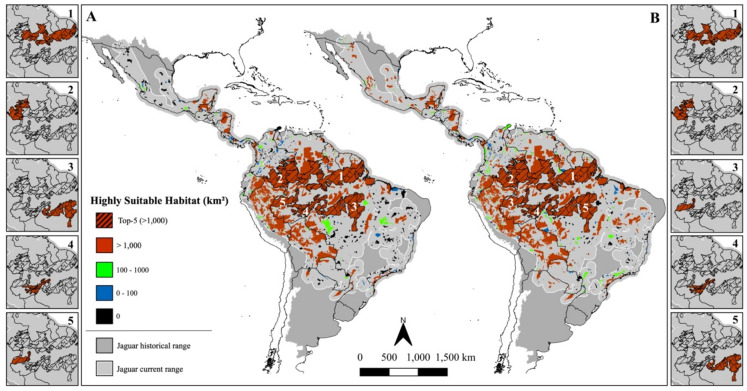
Protected Areas and Indigenous Lands (PAs) classified by the amount of highly suitable area for jaguars considering the range-based (**A**) and ecoregion-based scenarios (**B**). The hashed pattern and side maps underline the five main PAs in each scenario. Figure created using QGIS v3.36.0 (https://qgis.org).

When focusing on highly suitable habitats (upper quartile – top 25%), patterns varied depending on the analytical scenario (Fig. 2). In the range-based scenario, “Tropical & Subtropical Moist Broadleaf Forests” had a drastic domain, comprising 96.3% of all highly suitable habitat. This dominance was also evident within JCUs and PAs (Figs. 3A and 4A): the five units with the largest extents of highly suitable habitat were all located in the Amazon – a “Moist Broadleaf Forest”, together accounting for approximately 9.8 and 4.7 million km^2^ in JCUs and PAs, respectively (see Online Appendix S1 and S2 for detailed values). These findings emphasize the significance of large, continuous wild areas for jaguar conservation.

In the ecoregion-based scenario, however, the picture became more geographically diverse (Fig. 2). Although “Moist Broadleaf Forests” still held the majority (58.9%), other ecoregions gained prominence: “Tropical & Subtropical Grasslands, Savannas & Shrublands” and “Tropical & Subtropical Dry Broadleaf Forests”, for instance, accounted for 20.7% and 10.9% of the highly suitable habitat, respectively. This shift was mirrored in JCUs and PAs (Figs. 3B and 4B). JCUs in the Pantanal and Gran Chaco biomes ranked among the top five, and PAs of known conservation value in Mexico, the Cerrado, and Caatinga also featured prominently, each containing over 1000 km^2^ of highly suitable habitat.

When suitability was assessed as a proportion of each JCU or PA’s area rather than absolute amount, similar trends emerged. In the range-based scenario, Amazonian units again stood out (Supplementary Fig. S4A and S5A), along with some in the Mayan Forest and Central America. In the ecoregion-based scenario, high-proportion areas were also identified in the Pantanal, Gran Chaco, Cerrado, Caatinga, and Mexico’s coastal regions (Supplementary Fig. S4B and S5B), offering a more regionally balanced view.

At the national level, Brazil holds the largest share of the jaguar’s current range – about 29.8 million km^2^ or 50.7% – followed by Mexico (9.2%) and Colombia (7.5%) (Table 3). Brazil also contains the largest extent of highly suitable habitat under both scenarios: 7.9 million km^2^ in the range-based analysis and 6.2 million km^2^ in the ecoregion-based analysis. In several other countries, however, the difference between scenarios is more pronounced, highlighting the regional importance of habitats that may be overshadowed when the analysis is dominated by “Tropical and Subtropical Moist Broadleaf Forests” – primarily the Amazon. Regarding protection, an average of 54.3% of highly suitable habitats is under formal protection in the range-based scenario, and 43.4% in the ecoregion-based scenario.

**Table 3:** Country-wise assessment of the estimated amount of highly suitable habitat (top quartile of suitability) for Jaguars (*Panthera onca*).

Country	Current range (proportion)	Highly suitable area			
		Range-based [km^2^] (percentage)	Proportion protected (%)	Ecoregion-based [km^2^] (percentage)	Proportion protected (%)
Argentina	1,741,486 (2.97)	29,095 (1.7)	76.2	502,426 (28.9)	17.8
Belize	93,528 (0.16)	46,584 (49.8)	43.9	31,600 (33.8)	39.5
Bolivia	3,684,688 (6.27)	392,521 (10.7)	59.9	1,389,385 (37.7)	55.0
Brazil	29,792,722 (50.7)	7,849,538 (26.3)	62.1	6,232,107 (20.9)	54.0
Colombia	4,409,554 (7.51)	1,156,196 (26.2)	81.8	868,661 (19.7)	72.2
Costa Rica	209,228 (0.36)	7,048 (3.4)	78.2	8,043 (3.8)	53.3
Ecuador	821,461 (1.40)	271,792 (33.1)	87.1	258,322 (31.4)	86.3
French Guiana	336,117 (0.57)	191,019 (56.8)	41.7	48,008 (14.3)	33.7
Guatemala	449,440 (0.77)	48,764 (10.8)	99.0	26,295 (5.9)	90.4
Guyana	851,206 (1.45)	447,481 (52.6)	18.3	241,653 (28.4)	27.4
Honduras	444,194 (0.76)	59,430 (13.4)	75.4	94,116 (21.2)	44.0
Mexico	5,412,138 (9.21)	402,622 (7.4)	34.0	1,303,492 (24.1)	18.9
Nicaragua	527,923 (0.90)	82,327 (15.6)	53.8	77,836 (14.7)	32.2
Panama	295,251 (0.50)	22,867 (7.7)	60.7	8,002 (2.7)	63.4
Paraguay	1,680,725 (2.86)	62,217 (3.7)	38.4	711,125 (42.3)	11.4
Peru	3,491,372 (5.94)	2,297,798 (65.8)	55.8	1,716,467 (49.2)	56.9
Suriname	587,823 (1.00)	243,411 (41.4)	17.5	93,836 (16)	19.1
United States	334,762 (0.57)	0 (0)	0	42,637 (12.7)	18.6
Venezuela	3,572,203 (6.08)	970,291 (27.2)	47.8	923,865 (25.9)	29.9

“Current range” refers to the area (km^2^) of the jaguar’s current range within each country, while “Proportion” is the percentage this range represents relative to the total area of the current range. “Highly suitable area” corresponds to the area (km^2^) classified as highly suitable habitat (top quartile of suitability) per country. “Range-based” refers to the suitable habitat identified when the entire current range was analysed as a single unit, whereas “Ecoregion-based” reflects the suitable habitat identified when each ecoregion was analysed separately. “Percentage” indicates the share of suitable habitat relative to the total current range area within each country. “Proportion Protected” shows the percentage of this suitable habitat that overlaps with protected areas, for both the Range- and Ecoregion-based approaches.

## Discussion

The jaguar-habitat interactions found in our model demonstrated a trend of avoidance of anthropogenic features whilst favouring features associated with vegetation productivity and proximity to water. Such a pattern has been reported for the species at different geographic scales^30,34,38,39^. Jaguars are proficient swimmers and can even catch fish or caimans in the water^40^. In addition, they often use riverbanks for movement and hunting^41,42^, explaining their strong selection for water in our model. Similarly, productive habitats tend to have higher carrying capacity, benefiting species diversity and ultimately, carnivores^43^. Conversely, anthropogenic variables, such as human modification, human population density, livestock, defaunation, burned areas, and forest degradation, are mostly related to highly transformed regions and their surroundings, hence the avoidance found. For instance, while livestock can attract jaguars at a local scale by providing an accessible source of food^44^, suggesting a positive selection, the negative association found in our model likely reflects landscape-scale processes. Specifically, it captures the widespread conversion of natural habitats into pastures for extensive cattle grazing, rather than a direct avoidance of livestock itself. Jaguars were also negatively affected by topographic relief, higher surface temperature and higher seasonality in temperature. The general tendency to avoid seasonal climates indicates a decline in habitat suitability as distance from the equator increases, agreeing with a range-wide prediction of population density^34^. Meanwhile, jaguar avoidance of warmer surface temperatures is likely due to physiological constraints, as big cats have a limited capacity for thermoregulation^45^. Regarding topography, our model indicated that jaguars prefer low altitudes and valleys. This corroborates previous studies and the common understanding that high mountain ranges, such as the Andes, are probably barriers for the species^3,46^. Nevertheless, jaguars are known for their behavioural plasticity in adapting to varying conditions^38^. For instance, local studies in the Caatinga biome reported preference towards higher elevation^47,48^, while others in three different biomes of Mexico reported aversion to high altitudes, with jaguars using areas characterised by moderate slopes^17,49,50^. This variation highlights the complexities and limitations associated with predicting habitat use across the species’ distribution. Therefore, we reiterate that the patterns described here represent general trends, and local contexts should always be considered.

Jaguar responses to landscape features depended largely on the scale of effect, with interactions occurring at different spatial scales for each covariate. This scale-dependency in species-habitat relationships has long been known to science^51–53^, and widely described in carnivore habitat selection^51–53^. Our results corroborate this general pattern, although no clear selection for coarse scales was found as described in previous studies with the two large felids of the continent^16,54^.

The validation tests, along with the comparisons to previous estimates indicated that our model successfully predicted jaguar habitat suitability. For instance, the strong correlation between our predicted suitability surface and independent density estimates suggests that areas classified as highly suitable are indeed likely to support larger jaguar populations. This reinforces the model’s value for conservation planning across the species’ range. Building on this, we explored two complementary scenarios for categorizing habitat suitability – one based on the entire range and another stratified by ecoregion – to better understand both large-scale and region-specific conservation priorities. The range-based scenario highlighted the dominance of the Amazon, followed by the Mayan Forest, as hosting large expanses of highly suitable habitat. These two regions have previously been identified as key strongholds for jaguar conservation^4,34^, and our findings reinforce their importance. In contrast, the ecoregion-based approach, revealed additional high-value areas elsewhere, such as the Pantanal, Gran Chaco, Caatinga, and parts of Mesoamerica, underscoring the importance of regional context when setting conservation priorities. This divergence likely stems from the disproportionate extent of continuous natural habitat in the “Moist Broadleaf Forests” ecoregion – particularly the Amazon – which increases its relevance in the range-based scenario. Although the “Moist Broadleaf Forests” still contain a significant portion (59%) of highly suitable habitat in the ecoregion-based scenario, this approach also highlights other critical regions for the species, offering more a balanced insight for conservation planning.

These regional differences in habitat suitability become particularly evident when examining conditions across specific biomes and subregions under the ecoregion-based scenario. Along Mexico’s coasts, for instance, our model identifies several patches of highly suitable habitat – many of them with over 1000 km^2^ under formal protection (Fig. 4B). Nevertheless, our predictions suggest these habitats are more isolated than previously proposed^4,34^. While the Pacific coast has been considered a continuous corridor for jaguars, our results indicate possible gaps, particularly between the Chiapas region and the mangroves around Nayarit (Fig. 1a). Meanwhile, Atlantic coastal habitats appear to be highly fragmented, further emphasizing the importance of protection and connectivity efforts in the region. A similar pattern emerges in Central America, where jaguar habitat is highly fragmented across six countries, despite an average of 54% of the region’s highly suitable habitat being formally protected (Table 3). Consistent with previous estimates^55^, our model indicates at least three areas of very low suitability that may be isolating jaguar populations – along the Guatemala-Honduras border, in central Costa Rica, and in central Panama (Fig. 1b–d). Central America is key for maintaining jaguar population connectivity across their range. However, it faces alarming levels of forest loss and degradation^56^, and early signs of genetic isolation have already been detected in local populations^57^, underscoring the urgent need for continued monitoring and targeted conservation action.

In South America, the scenario is equally complex, with habitat suitability shaped by an intricate mosaic of biological and sociopolitical conditions. In the Darién Gap – at the edge between Central and South America – our model indicates favourable conditions for jaguars, consistent with previous predictions^58^. Nevertheless, the region is highly unstable due to the intense human migration and narcotrafficking activity^59,60^. The Llanos wetlands spanning Colombia and Venezuela revealed extensive areas of highly suitable habitat in our model, particularly across central regions bordering the Amazon rainforest – similarly to recent occupancy predictions for the biome^61^. Since European colonization, cattle ranching has dominated land use in the region, contributing to habitat loss and increased human–jaguar conflict. Today, only 14% of the jaguar’s current range in the Llanos is formally protected^61^. Despite these pressures, several independent studies have reported relatively high jaguar densities across the biome^61–63^, highlighting the Llanos’ importance for the species and its potential as an ecological corridor linking southern and northern jaguar populations in South America. In the other major South American freshwater wetland, the Pantanal, a similar scenario unfolds with high jaguar population densities frequently reported despite the dominance of privately owned cattle ranches, occupying approximately 90% of the biome^64–66^. In recent years, however, the biome has faced increasing threats from wildfires, exacerbated by extreme droughts and expanding land conversion^67,68^. Several of these fires occurred in areas identified as highly suitable for jaguars – particularly in the northern and central portions of the biome – indicating a disproportional impacting to the species in the region^67^. Our models indicate that the highly suitable habitats of the Pantanal are directly connected to those of the Gran Chaco along its western borders, spanning Brazil, Bolivia, Paraguay, and Argentina. This forms the second-largest expanse of continuous suitable habitat for jaguars after the Amazon (Fig. 2). Although protected areas are sparse across the Gran Chaco – covering only about 9% of the biome – nearly all of them encompass more than 1000 km^2^ of highly suitable habitat (Fig. 4). However, the region is undergoing rapid human encroachment, and much of its jaguar habitat is under significant pressure from habitat loss and overhunting^69^. This is particularly concerning given that jaguar populations in the Gran Chaco are generally small^70,71^, further increasing their vulnerability.

Three other key biomes for jaguars also warrant attention: the Atlantic Forest, Caatinga and Cerrado. The Atlantic Forest is classified as a “Moist Broadleaf Forest” and therefore presents larger extents of highly suitable habitat under the range-based scenario (Fig. 2A). As previously predicted^21^, the largest highly suitable areas are concentrated in the Serra do Mar region (Fig. 1e) and the Green Corridor of Misiones (Fig. 1f). While these patches offer hope for jaguar recovery, the biome’s extreme fragmentation poses a major challenge. Signs of declining genetic diversity have already been detected in the region’s jaguar populations^72^, and our models indicate that only the two aforementioned regions contain PAs with more than 1000 km^2^ of highly suitable habitat (Fig. 4A). In the Caatinga, our model identified the central-western region as the main stronghold for jaguars, aligning with prior studies^48^. The jaguar population in this biome is considered Critically Endangered, and signs of reduced genetic diversity have also been reported in the region^73^. Previous studies suggest that the biome’s main potential connection to other populations is likely through the Cerrado along its western edge^18,74^. Although our study did not explicitly assess connectivity, our suitability predictions point in the same direction with higher levels of suitability around *Serra das Confusões* National Park (NP) and a protected area complex (*APA Dunas e Veredas do Baixo-Médio São Francisco* and *Boqueirão da Onça* APA and NP) – both encompassing areas with more than 1000 km^2^ of highly suitable habitat (Fig. 4B). As for the Cerrado, our predictions identified five main areas of continuous highly suitable habitat in the west and central-northern regions (Fig. 1g–k), partially aligning with previous studies^18,75,76^. Four of these areas (Fig. 1h–k) lie within the MaToPiBa region (an acronym for the Brazilian states of Maranhão, Tocantins, Piauí and Bahia), which is under intense anthropogenic pressure as it represents the current frontier of agricultural expansion^77,78^. Despite these growing threats, the Cerrado still retains approximately half of its natural vegetation^79^, underscoring its importance for jaguar conservation. Owing to its central position within the jaguar’s range, the Cerrado also functions as a crucial ecological corridor connecting multiple biomes – a role reflected in the spatial distribution of highly suitable areas identified by our models.

As the biome-level patterns illustrate, highly suitable jaguar habitats are distributed across a mosaic of land management types. To better understand the protection status of these areas, we examined their overlap with JCUs, PAs, and non-designated lands. JCUs, while identified by experts as areas of high conservation relevance, do not carry formal political designation and thus have limited practical effect for the species conservation^15^. Nonetheless, our model suggests that JCUs encompass a substantial share of priority areas, containing 76.7% and 62.2% of highly suitable habitats in the range-based and ecoregion-based scenarios, respectively. However, the assessment also reveals reduced suitability in smaller, isolated JCUs, underscoring the need for targeted monitoring and support for these areas. The same holds true for PAs, despite their evident effectiveness in securing suitable habitats for jaguars. Roughly half of all suitable habitats fall within PAs’ boundaries regardless of how they were assessed – total summed suitability (54%) or highly suitable areas (range-based: 59% and ecoregion-based: 47%). A strong body of literature already shows the key role that protected areas and Indigenous Lands have in safeguarding natural habitats and biodiversity^80,81^ and our study reinforces that. Still, conservation efforts should also extend beyond formally designated areas, as significant portions of highly suitable habitat remain unprotected. One prominent example is the extensive area of non-designated public lands in Brazil, which – despite covering only 4% of the species’ current range (about 63.5 million hectares) – contain nearly 10% of the predicted suitable habitat under both scenarios assessed. These lands also account for 50% of all deforestation in the Amazon^82^ – something that could be greatly reduced by designating them for legal protection or sustainable use. Such actions would strengthen the PA network in the Amazon, ensuring protection of large continuous natural areas, benefiting not only jaguars but the entire ecosystem.

In the national level assessment, we calculated the area covered by highly suitable habitat for jaguars as well as how much of it is formally protected. Overall, countries encompassing the Amazon biome totalled the largest areas of suitable habitat. This pattern is particularly pronounced under the range-based scenario, with Brazil, Peru, Colombia, and Venezuela leading the list – each containing between 7.8 and 1.0 million km^2^ of highly suitable habitat. As described before, though, under the ecoregion-based scenario this pattern becomes more geographically distributed. Peripheral countries at the edges of the jaguar’s current range see a marked increase in the proportion of their territory identified as highly suitable. For instance, when considering the percentage of highly suitable habitat relative to each country’s current range, Argentina, Paraguay, and the United States shift from having almost no suitable area in the range-based scenario to 29%, 42%, and 13%, respectively, in the ecoregion-based scenario. Considering formal protection of the highly suitable habitats, the average protection was relatively high regardless of the scenario considered – 54.3% in the range-based scenario and 43.4% ecoregion-based scenario. As jaguars require large areas of natural habitat to thrive, implementing new PAs is a strategy that tends to benefit a range of other species in the habitat - the “umbrella” effect^83^. In this sense, our results produce a good overview and update of the species’ main habitats, providing a good starting point for conservation planning at a national level.

### Scope and limitations

Modelling exercises are simplifications of reality and, as such, are inherently imperfect. Our model aimed to identify suitable habitats across a vast and highly heterogeneous region – a challenging proposition even with a dataset as diverse as ours. Although we combined an impressive dataset of 172 monitored jaguars, our dataset was uneven, with areas such as the eastern and western Mexican coasts underrepresented. This uneven sampling prevented us to fully evaluate individual and regional variations across our study area (e.g., ecoregion-specific models subsequently integrated into a single ensemble prediction). To account for regional and individual variability, we included jaguar identity as a random effect, partially isolating such differences. Nonetheless, adding more individuals, particularly from underrepresented regions, would certainly benefit the model.

Our dataset had great variability in interval frequency of the GPS-locations; therefore, it was not possible to predict habitat use through more refined methods, such as step and path selection functions. Nevertheless, all validations showed our predictions were highly accurate. This indicates that our approach was capable of producing robust predictions, retaining the landscape features selected by the species and predicting spatial patterns of suitable habitat.

Finally, although our data were limited to areas where jaguars are currently found, we extrapolated predictions across the species’ historic range (as considered in this study). We recognize that extrapolating into such areas carries uncertainty, particularly because validation is not feasible. Therefore, predictions in these regions should be interpreted with caution. In our view, though, the broader suitability surface provides value as an exploratory exercise, potentially supporting field assessments and informing reintroduction and translocation initiatives – such as the ongoing efforts led by Rewilding Argentina in Iberá. Furthermore, generating a continuous suitability surface across the historic range benefits future analyses, including the development of resistance surfaces and connectivity modelling^10^.

We hope our model serves as a valuable tool for governments and international agencies in developing effective conservation policies. Anthropogenic pressure, in various guises, is undeniably threatening the remaining jaguar habitat and our study highlights large, continuous protected areas as keystone elements in the network of high-quality jaguar habitat across its range. Considering the species vulnerability to landscape and climate change^84^, we strongly advocate for the expansion of protected areas and Indigenous Lands, alongside the reinforcement of existing ones, to ensure the species’ long-term adaptability. Equally important, guaranteeing connectivity across the range is paramount to maintain healthy jaguar populations. As a strong response to these threats, we call for the immediate allocation of currently non-designated lands for conservation and sustainable use, reinforcing global commitments to wildlife protection.

## Methods

### Study area & Jaguar telemetry data

**Fig. 5:**
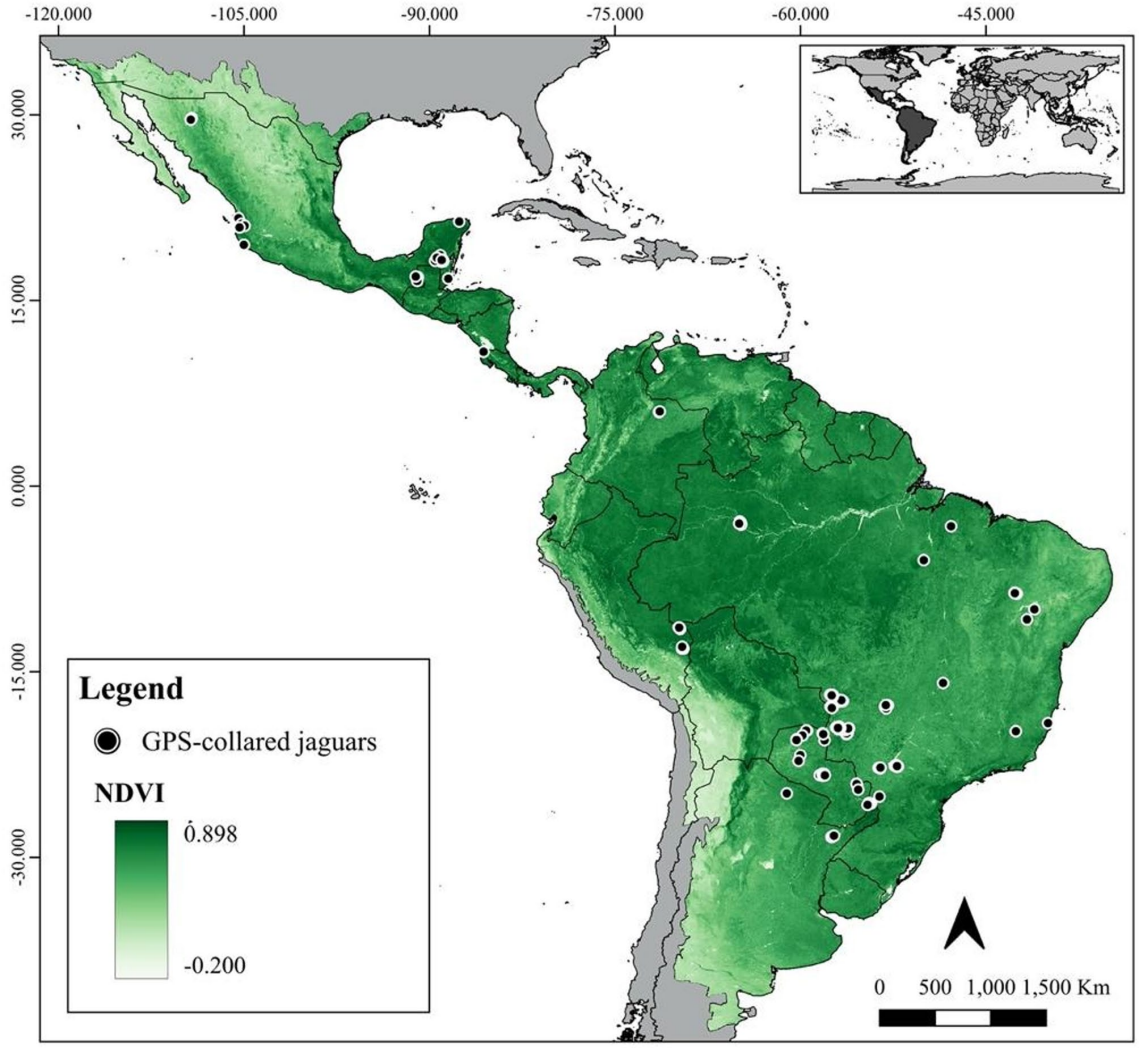
Reference sites of 172 GPS-collared jaguars (231,017 locations) monitored across the species range. The map depicts the mean normalized difference vegetation index (NDVI) in 2021 within the historical species range and the countries in grey. Figure created using QGIS v3.36.0 (https://qgis.org).

The study area covers the entire jaguar historical range (19,000,000 km^2^), from southwest US to central Argentina^2^ (Fig. 5). However, the precise boundaries of this historical range remain uncertain, leading to disagreements among experts regarding its true extent. These uncertainties are particularly notable at the edges, such as Baja California (Mexico) and the region near Península Valdés (Argentina), where historical jaguar presence is debated. For the purposes of this study, we consider the jaguar’s historical range based on the map previously produced^15^, which represents the extent of knowledge about the species’ status and distribution rather than a definitive record of past occurrence. This vast area exhibits great ecological heterogeneity, encompassing a diverse range of biological conditions reflected, for example, in the variety of habitats it harbours. It includes tropical rainforests, wetlands, dry forests, xeric forests, montane forests, temperate forests, savannahs, mangroves, and grasslands^15^.

We used GPS collar-tracking data from 172 jaguars (86 females and 86 males) captured and monitored in eight different countries: Argentina, Belize, Brazil, Colombia, Costa Rica, Mexico, Paraguay, and Peru (Fig. 5 and Supplementary Table S1). This dataset combines data from 117 jaguars originally published in a data paper^85^ and 55 additional individuals monitored under additional research projects led by co-authors of this study. All captures were conducted in accordance with the relevant institutional and governmental guidelines at the time, under permits obtained by the original project teams. No animals were captured specifically for this study. Jaguars were monitored across a wide range of ecological conditions, from arid regions of the xeric forests in the Northeast Brazil and the scrublands in the Northern Mexico, through tropical forests of the Atlantic Forest and the Mayan Forests, to the flooded habitats in the Paraguayan Chaco, Pantanal, and Amazonian floodplain forests. Since jaguars are capable of living in widely different ecological conditions, it is essential to represent habitat use across their range to produce accurate resource selection models^86^.

### Telemetry data processing

The 172 collared individuals (231,017 GPS locations) were monitored over different time periods across 25 years (from 1999 to 2023) with varying frequency intervals between the GPS-locations (Supplementary Table S1). To limit potential spatial bias, the locations of each individual were spatially rarefied, allowing only one location per 500 m pixel^87^. We applied a point selection function in which the ‘used’ habitat was sampled from the rarefied jaguar GPS-locations, and the ‘available’ habitat was sampled from randomly created background points. Background points did not overlap among themselves or with GPS-locations but were temporally and spatially delimited by the distribution of the GPS-locations of each jaguar.

Defining the size of the area available to create background points is crucial in resource selection functions as it directly affects model performance and covariate importance^88^. Essentially, background points should be a representation of the areas potentially available to an individual^89^. To define the available area for each individual, we used an estimate, in which home range size of mammalian species and their median dispersal distance is isometrically related by a multiple of seven^90^. We thus first averaged jaguar home range size for each biome, accounting for males and females separately^30^. Secondly, we extracted the square root of these home ranges and multiplied them by seven to calculate the potential dispersal distances per sex and biome (2nd order-selection^91^). These dispersal distances were used to create a buffer around GPS-locations of each jaguar, reflecting the available area for that individual. Within this buffer we randomly created 10 background points per each GPS-location, temporally matching them (e.g. for 100 GPS-locations from January 2020, 1000 background points would be created with the assigned January 2020 date). Temporal matching was important to correctly reflect the relationships between GPS-locations and background points when extracting information from the covariates. Finally, the dataset was divided into training (80% of presences) and validating data (20% remaining).

### Predictor variables

To model jaguar habitat suitability we selected a set of anthropogenic, ecological, climatic and geomorphologic variables known to influence wild felids with large home ranges^21,92,93^ (Table 4). The pervasive effects of human activities, such as habitat loss, fragmentation, direct persecution and depletion of prey populations are among the main threats to jaguars^3^. To account for these threats, we used the following variables: the global human disturbance index, human population density, night lights, roads, defaunation index, forest degradation, fire, and livestock density. For roads, we considered trunks, motorways, primary and secondary classes, excluding small unpaved classes because they may have opposite effects on jaguars – unpaved roads could be used as travel routes, while paved ones tend to be avoided^27,94,95^. To calculate the defaunation index, we used the dataset and approach of a previous study^96^ and pruned it to our study area. For the livestock density covariate, we combined the layers of cows (FAO^97^), goats (FAO^98^), and sheep (FAO^99^) as all three species tend to graze in open spaces and can be attacked by jaguars. Among ecological covariates, we tested: normalized difference vegetation index (NDVI), gross primary production (GPP), percentage of tree cover and canopy height. To describe climatic variability across the jaguar range, we selected land surface temperature, mean annual temperature, temperature seasonality, mean annual precipitation and precipitation seasonality. As our study area has significant variation in topography and soil composition, both known to influence jaguars and other large carnivores^93,100^, we chose elevation, topographic position index (TPI), compound topographic index (CTI), roughness, soil organic carbon, cation exchange capacity (CEC), clay, sand, nitrogen, and water surface. CTI, or wetness index, identifies the potential for water accumulation and flow^101^. Roughness describes terrain ruggedness while TPI assesses the relative position of a focal point in comparison to its surroundings, identifying if the point is in a flat area, a ridge or valley for example^102,103^. As for soil composition, CEC represents soil capacity to hold/exchange ions, which directly affects soil fertility^104^. All predictors were incorporated in numeric form – 28 as continuous variables and one (night lights) as binary – and used as untransformed gradients in the model. Roads, the only predictor originally provided as a vector layer, was rasterised into a binary layer. All the layers were resampled to 500 m as a reasonable trade-off between the size of the area considered in this analysis and jaguar movement patterns. A summary of original format, units, and final data structure for each predictor is provided in Table 4.

**Table 4:** Data used to derive covariates tested in the Jaguar habitat suitability model.

Category	Covariates	Resolution (m)	Year	Data type	Source
Anthropogenic	Global Human Modification	1000	2016	Continuous (0 to 1)	^125^
	Human Population Density	100	2002–2013^a^	Continuous (0 to 24,218)	^126^; www.worldpop.org
	Night Lights	1000	1992–2020	Continuous (0 to 63)	^127^
	Roads	Shapefile	2022	Binary (0/1)	OSM, www.openstreetmap.org
	Defaunation Index	500	1993–2019^b^	Continuous (0 to 1)	^96^
	Forest Degradation	30	1999–2021	Continuous (0 to 1)	^128^; EC JRC
	Fire	500	2000–2022	Binary (0/1)	^129^
	Livestock Density	1000	2005	Continuous (0 to 13,958)	^97–99^ FAO
Biological	Normalized Difference Vegetation Index (NDVI)	500	2000–2022	Continuous (−1 to + 1)	^130^; NASA EOSDIS Land Processes DAAC
	Gross Primary Production (GPP)	500	2000–2022	Continuous (0 to 0.3)	^131^; NASA EOSDIS Land Processes DAAC
	Percentage of Tree Cover	250	2000–2020	Continuous (0 to 1)	^132^; NASA EOSDIS Land Processes DAAC
	Canopy Height	30	2019	Continuous (0 to 36)	^133^; NASA GEDI
Climatic	Land Surface Temperature	1000	2000–2022^c^	Continuous (−0.4 to 48.9)	^134^; NASA EOSDIS Land Processes DAAC
	Mean Annual Temperature	1000	1979–2013^d^	Continuous (−87 to 293)	^135^; CHELSA-BIOCLIM+
	Temperature Seasonality			Continuous (147 to 8,283)	
	Mean Annual Precipitation			Continuous (12 to 11,089)	
	Precipitation Seasonality			Continuous (6 to 250)	
Geomorphological	Elevation	90	2000	Continuous (−71 to 6292)	^136^
	Topographic Position Index (TPI)	90	2000	Continuous (−15 to 15.9)	^103^
	Compound Topographic Index (CTI)			Continuous (−4 to 10.2)	
	Roughness			Continuous (0 to 297)	
	Soil Organic Carbon	250	1960–2022^e^	Continuous (10 to 2,443)	^137^
	Cation Exchange Capacity (CEC)			Continuous (26 to 597)	
	Clay			Continuous (34 to 707)	
	Sand			Continuous (5.3 to 879.1)	
	Nitrogen			Continuous (152 to 12,222)	
	Water surface	30	1999–2020	Continuous (0 to 100)	^138^

^a^It uses census from 2002 to 2013 to create one raster. ^b^Authors combined mammal assemblage data covering this temporal range. ^c^We calculated the seasonal mean for all years of the range, producing one layer for each season. ^d^Mean of all years of the range. ^e^It aggregates soil data from before 1960 until today.

### Data analysis

Resource selection is inherently scale-dependent, and habitat use can be driven not only by local site characteristics but also by broader landscape context^12,105^. To account for this, we identified the best spatial scale for each tested covariate, following a multi-scale optimization approach (*sensu*^12,13^). For each covariate, we fitted a Gaussian kernel of varying width around presences and pseudo-absences, reflecting seven spatial scales: 0.5 km, 1 km, 2 km, 4 km, 8 km, 16 km, and 32 km. These scales were selected based on previous studies on jaguars^16^ and clouded leopards^106^. Whenever possible, covariates, GPS-locations and background points were temporally matched or assigned to the closest date. Thus fire, forest degradation, night lights, and percentage of tree cover were annually matched, while GPP and NDVI were seasonally and annually matched, and surface temperature and water surface were seasonally averaged from data ranging from 2000 to 2020. All other variables were used according to the date/year available. Spatial analyses were done in R v3.5.1^107^, QGIS v3.36.0^108^, and Google Earth Engine^109^.

Previous studies have shown that individual jaguars often behave differently, adapting according to the resources available in the landscape^30,110^. To account for this variability in individual behaviour, we applied conditional logistic regression (Generalized Linear Mixed Model - GLMM), accounting for jaguar ID as the random factor, to evaluate jaguars’ habitat use. For variable selection we adopted an approach identified by a comprehensive simulation^13^ as the best workflow for variables and scale optimization. Firstly, we ran mixed-effect conditional logistic models at each scale, followed by model selection based on Akaike Information Criterion corrected for small sample size (AICc), retaining the scale for each variable with the lowest AICc^111^. Once the optimal scales were defined, we used the minimum redundancy-maximum relevance (mRMR) technique to find the most complementary and relevant set of covariates, applying Spearman’s estimator to test covariates correlation^112^.

As a good balance between model complexity and prediction performance, we selected the 15 most relevant covariates at their optimal scales. In the final step, the remaining covariates were standardized, and we applied a multiple mixed-effect conditional logistic regression model (random factor: jaguar ID). To produce the suitability surface, we used the most recent layers of the covariates selected in the final model at their optimal scale. Because RSFs fitted to use–availability data estimate relative selection strength rather than absolute probability of occurrence, predicted values represent proportional selection^113^. Accordingly, the final suitability surface is visualized using a continuous low-to-high gradient rather than an absolute probability scale. For mapping and model validation, the prediction raster was classified into ten equal-frequency (quantile-based) bins to ensure consistency with the Boyce Index assessment, with linear colour interpolation applied solely to smooth visual transitions between classes. The covariates “surface temperature”, “water surface”, and “NDVI” had their seasonal values averaged also using the most recent date possible. Models were conducted in R v3.5.1^107^, with packages *mRMRe*^112^ and *lme4*^114^, and predictions across the whole range were generated with the package *terra*^115^.

### Model validation and comparison

We evaluated our suitability surface using three different datasets and two approaches. In the first approach, we used the 20% of our dataset that was set aside for validation to assess how much the model differed from random expectations applying the Boyce index - a method widely used to evaluate models developed based on presence-only data^116^. This method divides the prediction layer into bins (classes) based on intervals of predicted values – in our case 10 equal-frequency bins representing 10 quantiles – and measures the frequency of predicted and expected values for each class^116^. The Boyce index varies between − 1 and 1, with negative values indicating counter predictions, values close to zero a random model and values near 1 a highly accurate prediction^117^. In the second approach, we selected a completely independent dataset – jaguar occurrence records^118^ – and calculated the area under the Receiver Operating Characteristic (ROC) curve (AUC), computed with the *pROC* package in R^119^. We also identified the optimal classification threshold based on the point closest to the top-left corner of the ROC space to calculate model sensitivity and specificity. The jaguar occurrence records (presence-absence) were derived from several sources, such as camera trapping, line transects and interviews, and were filtered to include only data from the most recent five-year period available (2013–2018) (*N* = 2161).

Additionally, we compared our suitability surface with other predictions available in literature. Specifically, we compiled jaguar density estimates from literature by combining data from Jędrzejewski et al.^34^ with additional studies published since then. To minimize potential bias from differing density estimation methods, we included only estimates derived from spatial explicit capture-recapture models (SECR) (Supplementary Table S2; *N* = 80). For each location, we generated a buffer with an area of 100 km^2^ – the scale used in jaguar density estimates – and extracted the average suitability within each buffer to serve as the predictor variable in a linear regression analysis. Finally, to assess the consistency between different methodologies, we performed a Pearson correlation test between our suitability surface and the distribution model from Jędrzejewski et al.^34^.

### Conservation implications

To assess the conservation implications of our suitability surface, we evaluated the distribution of suitable habitats across non-designated lands (specific to Brazil), JCUs, PAs and countries. Non-designated lands are public areas that have not been officially assigned for specific uses, such as Indigenous Lands, protected areas, or agrarian reform settlements (Supplementary Fig. S1). In Brazil, these lands cover approximately 63.5 million hectares, primarily distributed across the Cerrado (savannahs) and Amazon biomes, and due to their unclear ownership, are highly vulnerable to illegal deforestation and land grabbing (*grilagem* in Portuguese)^82^.

In the following analyses, we used the most up-to-date layers of non-designated lands^120^, JCUs^121^ and PAs (including Indigenous Lands) (Supplementary Fig. S1). The PAs layer from the World Database^122^ was refined using a South American dataset^8^ and country-level information with the support of experts. During this process, adjacent PAs within the same country were merged and assessed as one single protected block. While we acknowledge that different PA categories have varying levels of law enforcement, our primary goal was to distinguish protected (implemented with whatever stringency) from non-protected areas, which was both more feasible and informative than evaluating each category separately. To be included in our assessment, PAs (or PA blocks) had to meet three criteria: they had to be larger than 100 km^2^, as jaguar are less likely to occur in smaller PAs^22^; located below 3000 m in elevation, since no confirmed jaguar records exist above this threshold^123^; and intersect the jaguar’s current range. As an estimate of current range, we combined the updated South America distribution^8^ with the WCS jaguar distribution layer for the remaining area (from Panama to South US ^124^). Finally, assuming potential spatial uncertainties in the data and the species’ high mobility (median dispersal distance: 111 km, calculated following Bowman et al.^90^), we applied a 100 km buffer around the estimated distribution. We considered this buffered area as the final jaguar’s current range and used it to filter PAs for our assessment – except for El Salvador, where jaguars are currently extinct (Supplementary Fig. S1).

To explore our results, we used two complementary approaches: one treating the suitability surface as continuous data, and another based on categorized values. In the first, we calculated the summed total predicted habitat suitability across the species’ current range and extracted the proportion of this total found within non-designated lands, JCUs, and PAs. In the second approach, we classified as highly suitable those areas falling within the upper quartile (top 25%) of the suitability surface. This classification was applied in two ways: first, across the entire current range (range-based scenario), and second, separately within each ecoregion (ecoregion-based scenario). In the range-based scenario, we quantified the amount of highly suitable habitat for the entire range as a unit, extracting then its amount within non-designated lands, JCUs, PAs, and countries. In the ecoregion-based scenario, we identified the highly suitability values within each ecoregion and then calculated how much of that highly suitable habitat fell within the same land categories. Because larger JCUs and PAs are more likely to contain more suitable habitat, we also calculated the proportion of highly suitable habitat relative to the area of each unit. Finally, to assess the importance of JCUs and PAs independently of size, we grouped them into five categories based on the percentage of highly suitable habitat they contain: 100–75%, 75–50%, 50–25%, 25–0%, and 0%.

## Supplementary Information

Below is the link to the electronic supplementary material.

Supplementary Material 1

Supplementary Material 2

Supplementary Material 1

## Data Availability

GPS telemetry data for 117 of the 172 jaguar individuals used in this study are publicly available via Morato et al. (2018)^82^. The remaining 55 individuals were provided by collaborators and remain under the stewardship of their respective research groups; these data are not publicly available due to ongoing research use and data-sharing agreements. However, access to these data may be granted upon reasonable request to the corresponding authors, pending approval from the original data providers. The resulting habitat suitability surface generated by this study will be made openly available on the Zenodo repository upon manuscript acceptance (currently accessible for peer review at: https://zenodo.org/records/15824344?token=eyJhbGciOiJIUzUxMiJ9.eyJpZCI6IjY3YTJjNzhjLTVmMzItNDFhZi04YmY1LTk0NTQzZmFkYjgyZSIsImRhdGEiOnt9LCJyYW5kb20iOiJiYTI3YTBjNDRhOGRkNjk3NWI1ZGI1OWEyMDRkYWU3NCJ9._5XjPvxWOw4azyxXv4Ww-eaoHm1FG54BexND5TEEsmBnBTahFRBpbdScnwD_8McXtH-eNHyVaqA6hoWbieufCQ).
