# Correction: Range-wide assessment of habitat suitability for jaguars using multiscale species distribution modelling

**DOI:** 10.1038/s41598-026-39595-0

**Published:** 2026-03-02

**Authors:** Guilherme Costa Alvarenga, Caroline C. Sartor, Samuel A. Cushman, Alexandra Zimmermann, Ana Carolina Srbek-Araujo, Ana Cristina Mendes-Oliveira, Bart Harmsen, Carlos De Angelo, Carolina Franco Esteves, Claudia B. de Campos, Daiana Jeronimo Polli, Diego F. Passos Viana, Diogo Maia Gräbin, Emiliano Donadio, Emiliano E. Ramalho, Esteban Payán, Fernando C. C. Azevedo, Francisco Palomares, George V. N. Powell, Gerardo Ceballos, Grasiela Porfirio, Heliot Zarza, Ivonne Cassaigne, Juliano A. Bogoni, Leonardo Sena, Louise Maranhão, Marcos Roberto Monteiro de Brito, Mathias W. Tobler, Øystein Wiig, Rebecca J. Foster, Ricardo Sampaio, Rodrigo Nuñez, Ronaldo G. Morato, Valeria Boron, Wener Hugo Arruda Moreno, Yadvinder Malhi, David W. Macdonald, Żaneta Kaszta

**Affiliations:** 1 Wildlife Conservation Research Unit (WildCRU), Department of Biology, University of Oxford, Life and Mind Building, South Parks Road, Oxford, OX1 3EL UK; 2 Grupo de Pesquisa em Ecologia e Conservação de Felinos na Amazônia, Mamirauá Institute for Sustainable Development (MISD), Estrada do Bexiga, nº 2584, Tefé, AM Brazil; 3 Department of Biological Sciences, Northern Arizona University, Flagstaff, AZ USA; 4 Programa de Pós-Graduação em Ciência Animal e Programa de Pós-Graduação em Ecologia de Ecossistemas, Universidade Vila Velha, Av. Comissário José Dantas de Melo, 21, Boa Vista, Vila Velha, Espírito Santo 29102-920 Brazil; 5 Laboratório de Ecologia e Zoologia de Vertebrados (LABEV/ICB), Universidade Federal do Pará, Belém, PA Brazil; 6 Panthera, 104 West 40th Street, 5th Floor, New York, NY 10018 USA; 7 Instituto de Ciencias de la Tierra, Biodiversidad y Ambiente (IBCIA), Universidad Nacional de Río Cuarto and National Scientific and Technical Research Council (CONICET), Ruta Nacional 36 Km 601, Río Cuarto, Argentina; 8 Institute for the Conservation of Neotropical Carnivores, Avenida Horácio Neto, 1030, Parque Edmundo Zanoni, Atibaia, SP Brazil; 9 Programa de Pós-Graduação em Ecologia e Conservação, Instituto de Biociências – Inbio, Universidade Federal de Mato Grosso do Sul, Campo Grande, MS 79070-900 Brazil; 10 Fundación Rewilding Argentina, Scalabrini Ortiz 3355, 1425 Buenos Aires, Argentina; 11 WCS Big Cat Program, New York, USA; 12 Departamento de Ciências Naturais, Universidade Federal de São João del Rei, São João del Rei, MG 36301-160 Brazil; 13 Department of Conservation Biology, Doñana Biological Station, CSIC, Avda. Américo Vespucio 26, 41092 Isla de la Cartuja, Seville Spain; 14 Wildlife Protection Solutions, 2501 Welton Street, Denver, CO 80205 USA; 15 Laboratorio de Ecología y Conservación de Fauna Silvestre, Instituto de Ecología, Universidad Nacional Autónoma de México, Ciudad Universitaria, Coyoacán, Ciudad de México, Mexico; 16 Instituto Homem Pantaneiro, Corumbá, Mato Grosso do Sul Brazil; 17 Departamento de Ciencias Ambientales, Universidad Autónoma Metropolitana, Unidad Lerma, CBS, Lerma de Villada, Mexico; 18 Primero Conservation, Box 158885935, Pinetop, AZ USA; 19 Centro de Pesquisa de Limnologia, Biodiversidade e Etnobiologia do Pantanal; Laboratório de Mastozoologia; Programa de Pós-graduação em Ciências Ambientais, Universidade do Estado de Mato Grosso-UNEMAT, Cáceres, MT 78217-900 Brazil; 20 Mamirauá Institute for Sustainable Development (MISD), Estrada do Bexiga, nº 2584, Tefé, AM Brazil; 21 Associação Onçafari, São Paulo, SP Brazil; 22 San Diego Zoo Wildlife Alliance, Conservation Science and Wildlife Health, 15600 San Pasqual Valley Road, Escondido, CA 92027 USA; 23 Natural History Museum, University of Oslo, POB 1172 Blindern, 0318 Oslo, Norway; 24 Centro Nacional de Pesquisa e Conservação de Mamíferos Carnívoros (CENAP-ICMBio), Estrada Municipal Hisaichi Takebayashi, 8600, Atibaia, SP 12952-011 Brazil; 25 Alianza Jaguar AC, Lab. Vida Silvestre, Fac. Biol. Universidad Michoacana, Morelia, Michoacán Mexico; 26 World Wide Fund for Nature (WWF) UK, The Living Planet Centre, Brewery Road, Woking, GU214LL UK; 27 Environmental Change Institute, School of Geography and the Environment, University of Oxford, South Parks Road, Oxford, UK; 28 Leverhulme Centre for Nature Recovery, University of Oxford, South Parks Road, Oxford, UK

Correction to: *Scientific Reports* 10.1038/s41598-025-30512-5, published online 24 December 2025

The original version of this Article contained an error in the order of the Figures. Figure 1 was published as Figure 2, Figure 2 was published as Figure 3, Figure 3 was published as Figure 4, Figure 4 was published as Figure 5, and Figure 5 was published as Figure 1.

The original Figures 1, 2, 3, 4 and 5 and accompanying legends appear below.

**Fig. 1:**
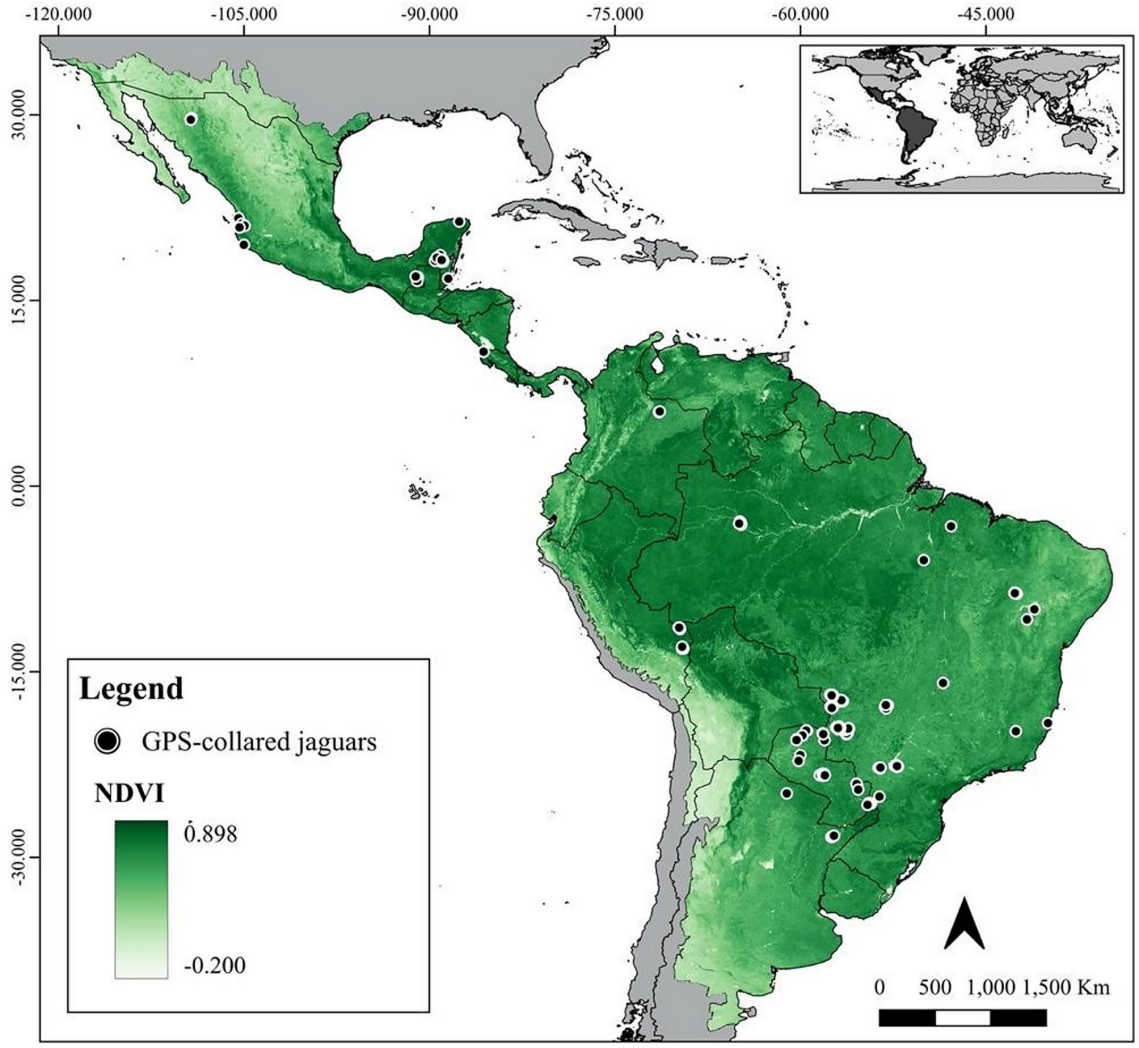
Predicted habitat suitability for jaguars (*Panthera onca*) across the species historical range. Letters mark specific regions of interest discussed in detail in the Discussion section. Figure created using QGIS v3.36.0 (https://qgis.org).

**Fig. 2:**
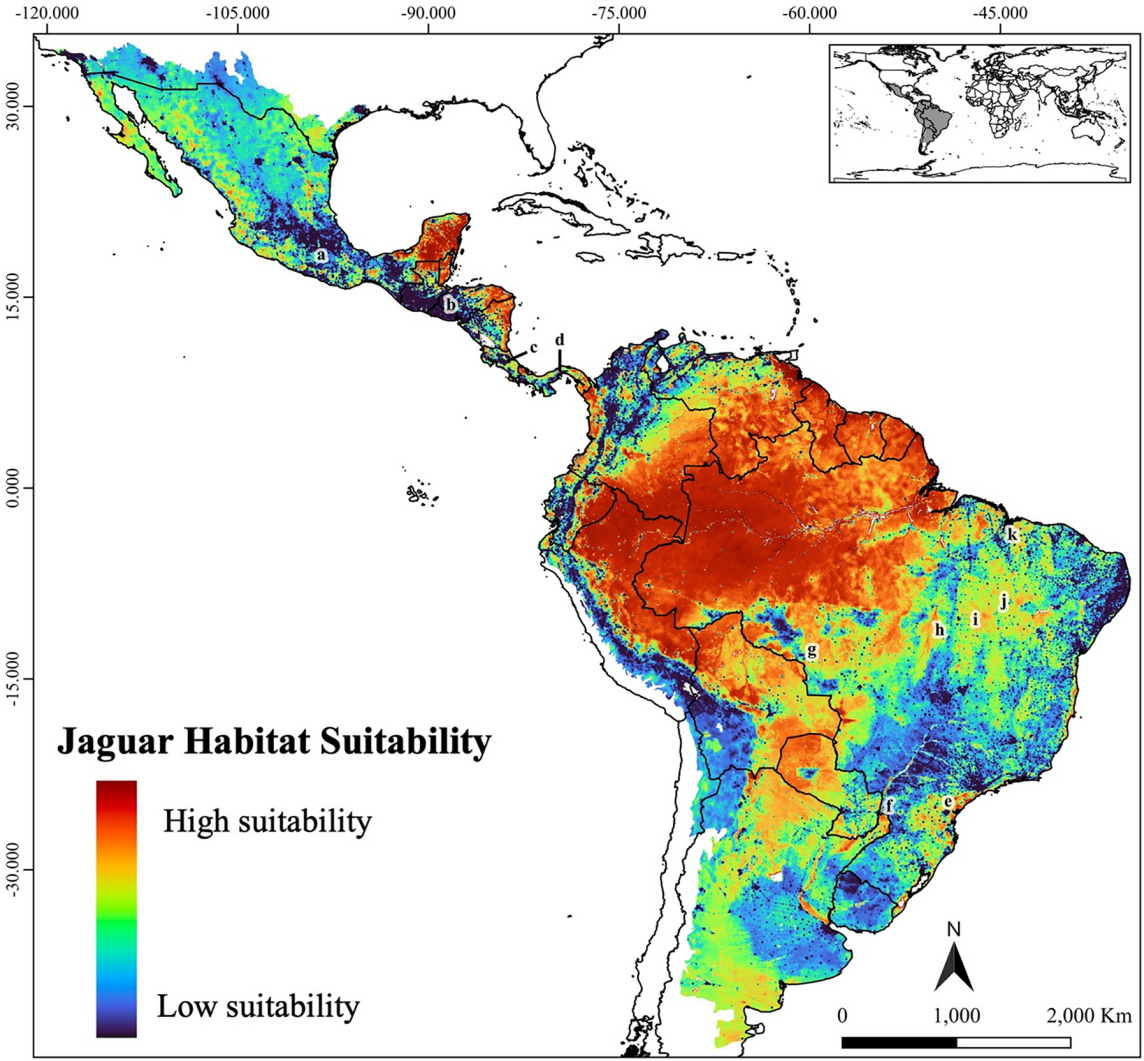
The jaguar highly suitable habitats considering the range-based (**A**) and ecoregion-based scenarios (**B**). Figure created using QGIS v3.36.0 (https://qgis.org).

**Fig. 3:**
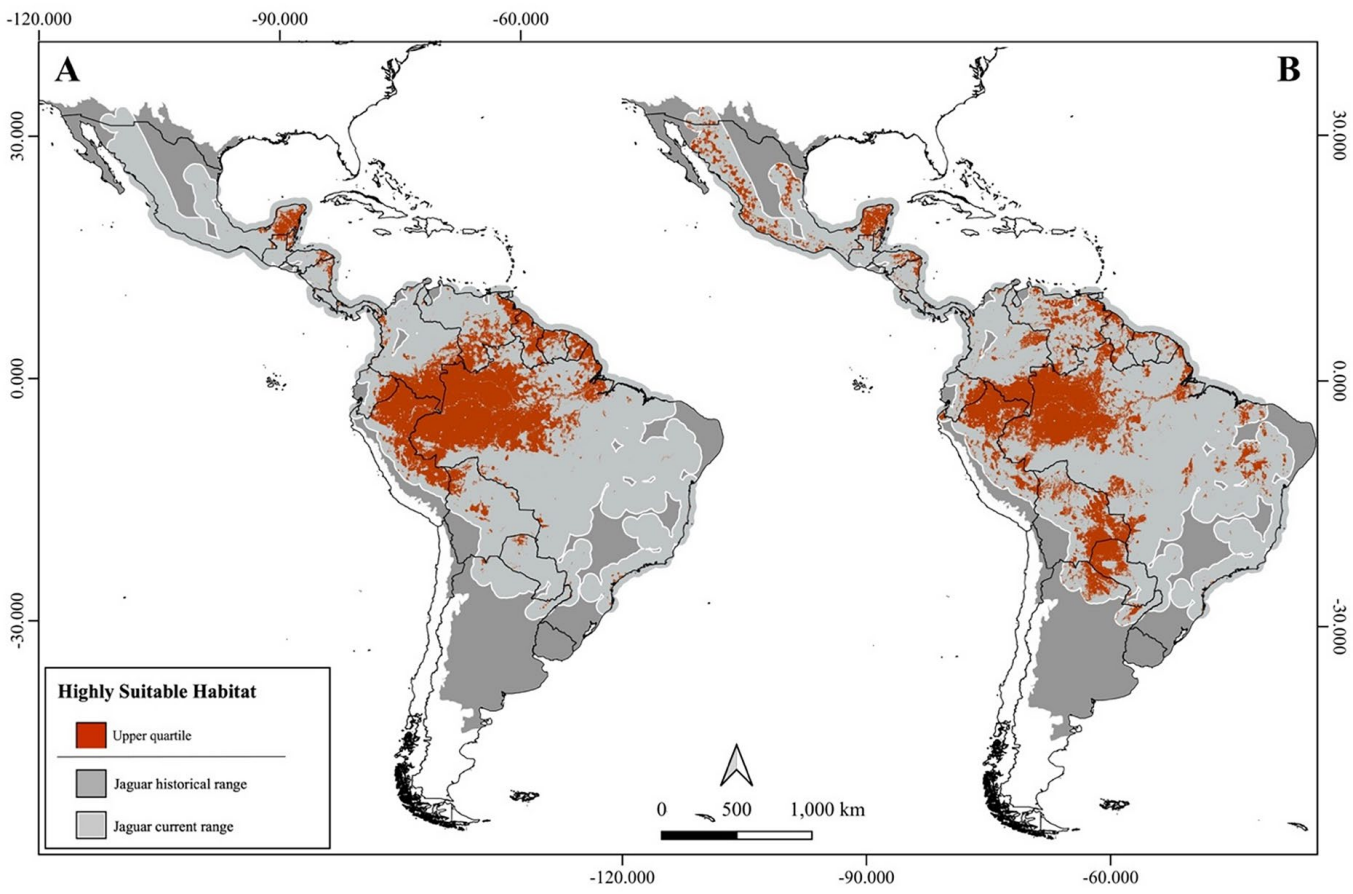
The Jaguar Conservation Units (JCUs) classified by the amount of highly suitable area for jaguars considering the range-based (**A**) and ecoregion-based scenarios (**B**). The hashed pattern and side maps underline the five main JCUs in each scenario. Figure created using QGIS v3.36.0 (https://qgis.org).

**Fig. 4:**
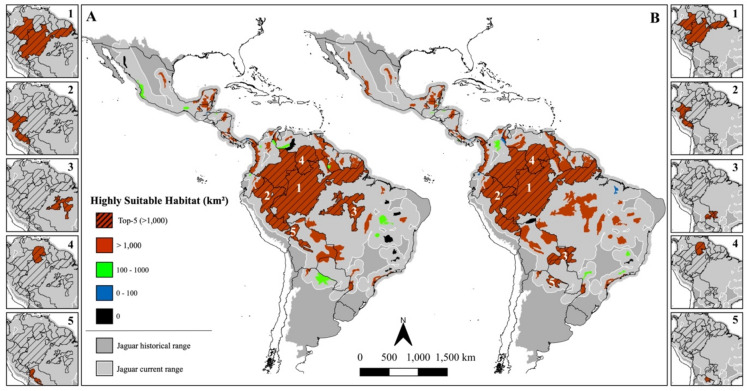
Protected Areas and Indigenous Lands (PAs) classified by the amount of highly suitable area for jaguars considering the range-based (**A**) and ecoregion-based scenarios (**B**). The hashed pattern and side maps underline the five main PAs in each scenario. Figure created using QGIS v3.36.0 (https://qgis.org).

**Fig. 5:**
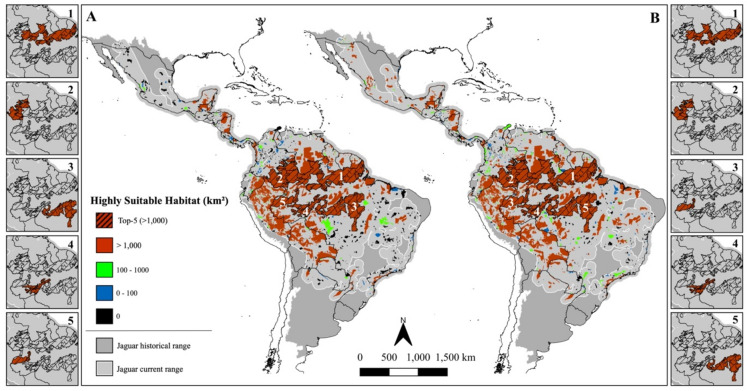
Reference sites of 172 GPS-collared jaguars (231,017 locations) monitored across the species range. The map depicts the mean normalized difference vegetation index (NDVI) in 2021 within the historical species range and the countries in grey. Figure created using QGIS v3.36.0 (https://qgis.org).

In addition, the Introduction section contained an error. Consequently,

“This effort represents a unique contribution to jaguar research and conservation, providing governments and stakeholders with essential information to guide conservation planning and policy (Table 1 and Figure 1).”

now reads:

“This effort represents a unique contribution to jaguar research and conservation, providing governments and stakeholders with essential information to guide conservation planning and policy.”

Additionally, in the Results section, under the subheading ‘Conservation implications’,

“This dominance was also evident within JCUs and PAs (Figures 3A and 5A): the five units with the largest extents of highly suitable habitat were all located in the Amazon – a “Moist Broadleaf Forest”, together accounting for approximately 9.8 and 4.7 million km^2^ in JCUs and PAs, respectively (see Online Appendix S1 and S2 for detailed values).”

now reads:

“This dominance was also evident within JCUs and PAs (Figures 3A and 4A): the five units with the largest extents of highly suitable habitat were all located in the Amazon – a “Moist Broadleaf Forest”, together accounting for approximately 9.8 and 4.7 million km^2^ in JCUs and PAs, respectively (see Online Appendix S1 and S2 for detailed values).”

“This shift was mirrored in JCUs and PAs (Figures 3B and 5B).”

now reads:

“This shift was mirrored in JCUs and PAs (Figures 3B and 4B).”

Furthermore, in the Discussion section,

“As previously predicted^21^, the largest highly suitable areas are concentrated in the Serra do Mar region (Figure 1e) and the Green Corridor of Misiones (Figure 2f).”

now reads:

“As previously predicted^21^, the largest highly suitable areas are concentrated in the Serra do Mar region (Figure 1e) and the Green Corridor of Misiones (Figure 1f).”

The original Article has been corrected.

