# Supplementary material for: Range-wide assessment of habitat suitability for jaguars using multiscale species distribution modelling
Source: Sci Rep. 2025 Dec 24;16:759. doi: 10.1038/s41598-025-30512-5 (PMC12780236; doi:10.1038/s41598-025-30512-5)
Supplement: Supplementary file 3 — Supplementary Material 1 [file 41598_2025_30512_MOESM3_ESM.docx]

**Supplementary**

**Table S1. Reference information of the 172 GPS-collared jaguars (*Panthera onca*) used in the study.**

| **Jaguar ID** | **Sex** | **Nº Locations** | **Locations Filtered** | **Tracked Period** | **Latitude** | **Longitude** | **Country** |
| --- | --- | --- | --- | --- | --- | --- | --- |
| 1 | F | 288 | 132 | 06/2010-03/2011 | -23.275652 | -58.116174 | Paraguay |
| 2 | F | 150 | 107 | 07/2010-12/2011 | -24.171145 | -55.433106 | Paraguay |
| 3 | M | 148 | 106 | 06/2009-09/2009 | -23.344435 | -58.450313 | Paraguay |
| 4 | F | 500 | 179 | 07/2011-12/2011 | -23.339263 | -58.138876 | Paraguay |
| 5 | F | 280 | 57 | 07/2009-09/2009 | -23.367736 | -58.1908264 | Paraguay |
| 6 | M | 983 | 221 | 07/2009-12/2009 | -23.232139 | -58.2325172 | Paraguay |
| 7 | M | 621 | 207 | 07/2011-11/2011 | -23.25648 | -58.077142 | Paraguay |
| 8 | M | 540 | 196 | 07/2009-07/2010 | -24.040429 | -55.486192 | Paraguay |
| 9 | F | 1018 | 177 | 06/2014-12/2014 | -23.326947 | -58.041257 | Paraguay |
| 10 | F | 1668 | 463 | 06/2014-05/2015 | -23.397239 | -58.185852 | Paraguay |
| 11 | F | 913 | 180 | 07/2014-07/2015 | -23.368049 | -58.039784 | Paraguay |
| 12 | F | 2681 | 259 | 12/2014-04/2015 | -16.9714833 | -57.3899431 | Brazil |
| 13 | M | 5040 | 108 | 12/2014-08/2015 | -16.9159483 | -57.4977108 | Brazil |
| 14 | M | 192 | 64 | 04/2012-04/2012 | -19.8744136 | -56.4016528 | Brazil |
| 15 | M | 1323 | 292 | 10/2013-01/2014 | -20.0301064 | -56.3210047 | Brazil |
| 16 | M | 3462 | 581 | 04/2011-08/2012 | -21.734897 | -60.0206985 | Paraguay |
| 17 | M | 537 | 187 | 03/2014-07/2014 | -17.808023 | -53.183055 | Brazil |
| 18 | M | 2314 | 177 | 11/2014-04/2015 | -16.9279125 | -57.4243325 | Brazil |
| 19 | F | 741 | 111 | 10/2011-01/2012 | -19.9582217 | -56.3169619 | Brazil |
| 20 | M | 5590 | 739 | 09/2015-06/2016 | -8.60977 | -42.6801 | Brazil |
| 21 | F | 327 | 139 | 08/2011-06/2012 | -24.067424 | -55.466862 | Paraguay |
| 22 | M | 4709 | 171 | 09/2014-05/2015 | -16.8864897 | -57.4038397 | Brazil |
| 23 | M | 572 | 38 | 09/2014-09/2014 | -16.8747392 | -57.5129536 | Brazil |
| 24 | M | 153 | 75 | 12/2011-03/2012 | -5.97859 | -50.040107 | Brazil |
| 25 | F | 3219 | 188 | 10/2012-08/2015 | -19.9448036 | -56.2427367 | Brazil |
| 26 | F | 5924 | 294 | 11/2014-06/2016 | 10.8596451 | -85.6343205 | Costa Rica |
| 27 | F | 560 | 86 | 09/2010-11/2013 | -17.3260076 | -56.7544166 | Brazil |
| 28 | F | 205 | 63 | 07/2010-10/2010 | -17.828947 | -57.40595 | Brazil |
| 29 | F | 67 | 30 | 07/2011-09/2011 | -17.8398802 | -57.3940782 | Brazil |
| 30 | F | 581 | 125 | 10/2011-11/2012 | -17.3293188 | -56.7126546 | Brazil |
| 31 | F | 103 | 36 | 10/2013-02/2014 | -17.3367322 | -56.7099957 | Brazil |
| 32 | F | 240 | 104 | 10/2012-04/2013 | -17.3793419 | -56.7819766 | Brazil |
| 33 | F | 133 | 68 | 10/2013-01/2014 | -17.3285701 | -56.7594096 | Brazil |
| 34 | F | 92 | 36 | 02/2003-07/2003 | -22.63708499 | -52.26445905 | Brazil |
| 35 | F | 17 | 15 | 08/2000-11/2001 | -22.59397636 | -52.25259646 | Brazil |
| 36 | F | 32 | 28 | 07/1999-07/2003 | -22.5901957 | -52.38519834 | Brazil |
| 37 | F | 211 | 90 | 07/2002-11/2002 | -22.63878999 | -52.12664972 | Brazil |
| 38 | F | 18 | 18 | 07/2002-08/2002 | -22.53828697 | -52.25666345 | Brazil |
| 39 | F | 326 | 187 | 02/2002-04/2005 | -22.75705316 | -53.68426924 | Brazil |
| 40 | F | 183 | 100 | 05/2004-12/2004 | -22.80383586 | -53.55541512 | Brazil |
| 41 | F | 4952 | 89 | 12/2014-08/2015 | -16.8612211 | -57.4243539 | Brazil |
| 42 | M | 7668 | 1039 | 02/2009-10/2009 | -25.637647 | -54.299678 | Argentina |
| 43 | F | 1512 | 556 | 01/2013-02/2013 | 29.74605 | -109.23039 | Mexico |
| 44 | F | 104 | 65 | 01/2012-09/2013 | 16.835904 | -91.042886 | Mexico |
| 45 | F | 39 | 14 | 08/2012-08/2012 | 16.889464 | -90.955664 | Mexico |
| 46 | F | 443 | 250 | 08/2012-11/2013 | 16.898824 | -91.083115 | Mexico |
| 47 | M | 636 | 329 | 01/2012-02/2013 | 16.579612 | -90.999996 | Mexico |
| 48 | M | 72 | 53 | 08/2012-12/2012 | 16.938643 | -91.120305 | Mexico |
| 49 | M | 979 | 280 | 01/2014-12/2015 | 21.3790875 | -87.6018119 | Mexico |
| 50 | M | 1361 | 218 | 11/2014-01/2015 | -8.6345197 | -42.7049775 | Brazil |
| 51 | M | 727 | 238 | 06/2010-12/2010 | -20.530443 | -58.061521 | Paraguay |
| 52 | F | 615 | 40 | 11/2014-12/2014 | -16.9132783 | -57.4112914 | Brazil |
| 53 | M | 300 | 148 | 09/2010-10/2013 | -17.265488 | -56.8139292 | Brazil |
| 54 | M | 129 | 21 | 07/2010-08/2010 | -17.8506281 | -57.4067729 | Brazil |
| 55 | M | 141 | 65 | 06/2011-10/2011 | -17.3527833 | -56.5766747 | Brazil |
| 56 | M | 109 | 59 | 07/2011-10/2011 | -17.8231802 | -57.5760012 | Brazil |
| 57 | M | 28 | 8 | 07/2011-08/2011 | -17.8351375 | -57.4037579 | Brazil |
| 58 | M | 159 | 74 | 01/2003-06/2003 | -22.67648014 | -52.19555839 | Brazil |
| 59 | M | 434 | 223 | 10/2011-12/2013 | -17.3239385 | -56.7097386 | Brazil |
| 60 | M | 705 | 187 | 10/2012-10/2013 | -17.3536055 | -56.7575736 | Brazil |
| 61 | M | 109 | 61 | 06/2013-08/2013 | -17.2932905 | -56.7014317 | Brazil |
| 62 | M | 156 | 106 | 05/2003-10/2003 | -22.62201182 | -52.20074942 | Brazil |
| 63 | M | 797 | 408 | 03/2005-04/2006 | -22.74154717 | -53.54126196 | Brazil |
| 64 | M | 1694 | 639 | 10/2012-12/2012 | 29.61717 | -109.31089 | Mexico |
| 65 | F | 1010 | 445 | 03/2014-10/2014 | -17.871376 | -53.103104 | Brazil |
| 66 | F | 53 | 38 | 02/2013-06/2013 | -25.6485772 | -54.4214814 | Brazil |
| 67 | F | 131 | 69 | 03/2014-06/2014 | -17.706925 | -52.936313 | Brazil |
| 68 | M | 1003 | 194 | 11/2011-12/2011 | -20.0268575 | -56.2552267 | Brazil |
| 69 | F | 3464 | 317 | 10/2013-05/2014 | -19.9262692 | -56.2414756 | Brazil |
| 70 | M | 1094 | 487 | 06/2002-06/2003 | -22.195088 | -60.160038 | Paraguay |
| 71 | F | 921 | 443 | 07/2003-07/2004 | -19.753344 | -59.480243 | Paraguay |
| 72 | M | 722 | 290 | 07/2003-07/2004 | -19.747937 | -59.551036 | Paraguay |
| 73 | M | 620 | 257 | 06/2004-09/2004 | -20.142131 | -59.851225 | Paraguay |
| 74 | F | 1300 | 185 | 08/2005-05/2006 | -19.971896 | -58.217231 | Paraguay |
| 75 | F | 1694 | 215 | 08/2005-08/2006 | -20.039304 | -58.165714 | Paraguay |
| 76 | F | 1610 | 616 | 06/2006-07/2007 | -20.440784 | -60.320772 | Paraguay |
| 77 | M | 1376 | 576 | 06/2006-05/2007 | -20.51578 | -60.33193 | Paraguay |
| 78 | F | 798 | 292 | 07/2008-07/2009 | -24.522229 | -55.34041 | Paraguay |
| 79 | F | 2300 | 201 | 04/2015-08/2015 | -19.9936717 | -56.2941214 | Brazil |
| 80 | M | 479 | 66 | 05/2010-07/2010 | -25.60912108 | -54.49638269 | Argentina |
| 81 | M | 10988 | 401 | 10/2013-05/2015 | -16.97737 | -57.3910972 | Brazil |
| 82 | M | 1335 | 576 | 05/2014-12/2015 | -17.96005 | -53.082933 | Brazil |
| 83 | M | 112 | 49 | 01/2012-03/2012 | -25.08117226 | -53.62542169 | Brazil |
| 84 | F | 4860 | 227 | 04/2013-01/2014 | -19.95198 | -56.3223003 | Brazil |
| 85 | M | 780 | 400 | 03/2014-02/2015 | -17.690413 | -53.107739 | Brazil |
| 86 | F | 1391 | 276 | 10/2013-01/2014 | -19.9611475 | -56.1977572 | Brazil |
| 87 | F | 413 | 32 | 05/2012-06/2012 | -19.9725481 | -56.2818497 | Brazil |
| 88 | F | 1296 | 116 | 10/2013-04/2014 | -16.8718285 | -57.5228518 | Brazil |
| 89 | M | 2432 | 402 | 09/2013-10/2015 | -15.9061 | -48.477581 | Brazil |
| 90 | F | 322 | 164 | 08/2012-04/2013 | -25.7531786 | -54.5845814 | Argentina |
| 91 | M | 88 | 33 | 01/2011-01/2012 | -16.894062 | -57.492675 | Brazil |
| 92 | F | 96 | 43 | 01/2011-01/2013 | -16.917908 | -57.487805 | Brazil |
| 93 | F | 784 | 144 | 12/2010-11/2011 | -3.050582 | -64.852288 | Brazil |
| 94 | F | 3837 | 244 | 11/2012-02/2014 | -3.054921 | -64.845133 | Brazil |
| 95 | F | 542 | 109 | 02/2013-07/2013 | -2.933995 | -64.834189 | Brazil |
| 96 | M | 230 | 125 | 01/2013-04/2013 | -3.03768 | -64.82531 | Brazil |
| 97 | F | 31 | 3 | 02/2013-02/2013 | -3.037074 | -64.843443 | Brazil |
| 98 | M | 61 | 26 | 01/2013-09/2013 | -3.043342 | -64.884696 | Brazil |
| 99 | M | 294 | 110 | 10/2013-11/2014 | -2.944293 | -64.940658 | Brazil |
| 100 | M | 1024 | 319 | 03/2014-08/2015 | -3.090892 | -64.813113 | Brazil |
| 101 | M | 404 | 207 | 08/2015-03/2016 | -19.710234 | -56.357117 | Brazil |
| 102 | F | 151 | 83 | 03/2016-05/2016 | -19.603761 | -56.211355 | Brazil |
| 103 | F | 20 | 12 | 06/2014-06/2014 | -19.571285 | -56.076128 | Brazil |
| 104 | M | 134 | 59 | 08/2015-06/2016 | -19.578162 | -56.151681 | Brazil |
| 105 | F | 2113 | 251 | 07/2008-10/2009 | -19.51600071 | -57.04450005 | Brazil |
| 106 | M | 227 | 131 | 09/2009-10/2011 | -19.42884891 | -56.91394335 | Brazil |
| 107 | M | 287 | 93 | 07/2008-12/2008 | -19.53291063 | -56.78271003 | Brazil |
| 108 | M | 481 | 197 | 07/2008-11/2008 | -19.46945048 | -56.86500405 | Brazil |
| 109 | F | 165 | 72 | 08/2008-10/2008 | -19.56692044 | -56.91692119 | Brazil |
| 110 | M | 166 | 93 | 02/2010-05/2010 | -19.49668019 | -57.12129559 | Brazil |
| 111 | F | 1758 | 188 | 07/2008-04/2011 | -19.43423998 | -56.86302315 | Brazil |
| 112 | F | 202 | 47 | 07/2008-10/2008 | -19.63714532 | -56.9650143 | Brazil |
| 113 | F | 709 | 128 | 08/2008-07/2009 | -19.47401862 | -56.94045639 | Brazil |
| 114 | F | 1645 | 191 | 06/2008-07/2009 | -19.47993711 | -57.08448275 | Brazil |
| 115 | F | 952 | 247 | 06/2008-03/2009 | -19.53442239 | -56.97638914 | Brazil |
| 116 | M | 3340 | 146 | 10/2015-04/2016 | -16.8496492 | -57.5967928 | Brazil |
| 117 | F | 2820 | 116 | 10/2015-02/2016 | -16.9078125 | -57.4846269 | Brazil |
| 118 | F | 451 | 175 | 01/2015-01/2016 | -3.078417 | -64.829936 | Brazil |
| 119 | M | 516 | 182 | 01/2015-07/2015 | -2.852821 | -64.908878 | Brazil |
| 120 | F | 843 | 137 | 03/2016-11/2016 | -3.042296 | -64.856324 | Brazil |
| 121 | M | 643 | 209 | 01/2016-10/2016 | -3.108568 | -64.817603 | Brazil |
| 122 | M | 835 | 275 | 02/2017-01/2018 | -3.03765 | -64.90765 | Brazil |
| 123 | M | 2622 | 269 | 03/2018-02/2019 | -3.062562 | -64.792863 | Brazil |
| 124 | M | 801 | 145 | 02/2020-05/2020 | -3.021761 | -64.964985 | Brazil |
| 125 | M | 4890 | 436 | 07/2019-07/2020 | -19.14391 | -39.99278 | Brazil |
| 126 | F | 4363 | 771 | 06/2019-02/2020 | -10.76774 | -41.69304 | Brazil |
| 127 | F | 1774 | 343 | 05/2013-05/2014 | 18.662806 | -89.131262 | Mexico |
| 128 | F | 1221 | 653 | 07/2001-03/2009 | 18.232041 | -88.898627 | Mexico |
| 129 | F | 166 | 125 | 03/2002-03/2003 | 18.31096889 | -88.95445333 | Mexico |
| 130 | M | 526 | 72 | 05/2009-06/2009 | 18.383318 | -89.300211 | Mexico |
| 131 | M | 1315 | 329 | 05/2009-07/2009 | 18.270961 | -89.256905 | Mexico |
| 132 | M | 295 | 207 | 04/2005-11/2005 | 18.1338 | -89.0362 | Mexico |
| 133 | F | 457 | 208 | 04/2005-05/2006 | 18.176021 | -88.888454 | Mexico |
| 134 | F | 307 | 121 | 04/2001-01/2002 | 18.295253 | -88.979485 | Mexico |
| 135 | M | 4831 | 871 | 01/2021-01/2023 | -24.8283769 | -61.1156142 | Argentina |
| 136 | F | 1290 | 190 | 05/2009-07/2009 | 18.324955 | -89.397159 | Mexico |
| 137 | M | 364 | 253 | 06/2002-05/2003 | 18.215324 | -89.558898 | Mexico |
| 138 | M | 1192 | 244 | 05/2009-09/2009 | 18.457128 | -89.412146 | Mexico |
| 139 | F | 245 | 141 | 04/2008-03/2009 | 18.231913 | -88.898713 | Mexico |
| 140 | F | 286 | 165 | 04/2005-08/2007 | 18.269887 | -89.02367 | Mexico |
| 141 | M | 2851 | 557 | 10/2012-03/2013 | -11.41943858 | -69.84469181 | Peru |
| 142 | M | 1618 | 378 | 09/2009-12/2009 | -11.43448933 | -69.77944624 | Peru |
| 143 | M | 189 | 132 | 11/2012-02/2013 | -11.5411808 | -69.7436494 | Peru |
| 144 | F | 903 | 168 | 10/2012-12/2012 | -11.4049256 | -69.85074 | Peru |
| 145 | F | 3848 | 290 | 09/2007-02/2008 | -13.12314509 | -69.59072353 | Peru |
| 146 | F | 3054 | 321 | 08/2007-12/2007 | -13.12049567 | -69.46562018 | Peru |
| 147 | M | 4905 | 584 | 07/2012-05/2013 | -11.42838766 | -69.82209262 | Peru |
| 148 | F | 697 | 98 | 09/2007-12/2007 | -13.05271558 | -69.54118693 | Peru |
| 149 | M | 4199 | 686 | 07/2007-02/2008 | -12.97864727 | -69.58116607 | Peru |
| 150 | M | 2122 | 171 | 01/2022-06/2022 | -17.925562 | -57.476751 | Brazil |
| 151 | M | 13243 | 1274 | 08/2017-03/2019 | -9.95224 | -41.09542 | Brazil |
| 152 | M | 287 | 80 | 02/2018-05/2018 | 5.98766 | -71.44483 | Colombia |
| 153 | F | 257 | 28 | 02/2018-05/2018 | 6.0383939 | -71.3843953 | Colombia |
| 154 | M | 2555 | 384 | 11/2019-06/2020 | -3.24246 | -47.83422 | Brazil |
| 155 | F | 2361 | 278 | 09/2017-07/2018 | -19.8004 | -42.59652 | Brazil |
| 156 | M | 2174 | 461 | 11/2017-11/2018 | -19.80304 | -42.59469 | Brazil |
| 157 | F | 43 | 25 | 07/2021-08/2021 | 20.9937908 | -105.2199662 | Mexico |
| 158 | F | 1257 | 73 | 11/2019-03/2020 | 21.6542017 | -105.4480192 | Mexico |
| 159 | M | 296 | 70 | 12/2015-03/2016 | 20.929646 | -105.297234 | Mexico |
| 160 | M | 309 | 41 | 04/2016-07/2016 | 21.1989024 | -105.2000204 | Mexico |
| 161 | M | 88 | 41 | 07/2017-08/2017 | 21.0400516 | -104.9517662 | Mexico |
| 162 | M | 94 | 37 | 05/2019-11/2019 | 20.9081558 | -105.3694981 | Mexico |
| 163 | M | 189 | 131 | 06/2015-05/2016 | 19.5015409 | -105.0139881 | Mexico |
| 164 | F | 2852 | 134 | 10/2021-01/2023 | -28.267087 | -57.338167 | Argentina |
| 165 | M | 2412 | 401 | 01/2022-01/2023 | -28.329787 | -57.437795 | Argentina |
| 166 | F | 1263 | 161 | 04/2021-11/2021 | -28.343423 | -57.509335 | Argentina |
| 167 | F | 676 | 99 | 10/2022-01/2023 | -28.337012 | -57.441685 | Argentina |
| 168 | F | 5183 | 450 | 01/2021-01/2023 | -28.243248 | -57.302054 | Argentina |
| 169 | M | 1248 | 318 | 06/2023-08/2023 | -8.691146 | -42.521022 | Brazil |
| 170 | F | 3057 | 426 | 09/2022-01/2023 | -8.647565 | -42.67408 | Brazil |
| 171 | M | 362 | 141 | 02/2015-02/2016 | 16.77688791 | -88.4860169 | Belize |
| 172 | M | 597 | 139 | 02/2015-10/2015 | 16.7671903 | -88.4881106 | Belize |
| **TOTAL** | **-** | **231,017** | **37,453** | **-** | **-** | **-** | **-** |

**Table S2. Jaguar population density estimates (Spatially Explicit Capture-Recapture – SECR) used for model comparison.**

| **ID** | **Date** | **Location** | **Latitude** | **Longitude** | **Density estimate** | **Average suitability** | **Study** |
| --- | --- | --- | --- | --- | --- | --- | --- |
| 1 | 2008-2011 | Amazon, Brazil | -9.82316 | -58.27086 | 3.03 | 0.075 | ^1^ |
| 2 | 2011 | Amazon, Guyana | 4.6024 | -58.7774 | 1.72 | 0.298383 | ^2^ |
| 3 | 2013-2014 | Amazon, French Guiana | 5.2024 | -52.8186 | 3.22 | 0.132664 | ^3^ |
| 4 | 2007-2009 | Amazon, Ecuador | -1.676 | -76.0065 | 5.44 | 0.445306 | ^4^ |
| 5 | 2007-2009 | Amazon, Ecuador | -0.7043 | -76.0044 | 1.49 | 0.555279 | ^4^ |
| 6 | 2007-2009 | Amazon, Ecuador | -1.00503 | -77.07275 | 0.89 | 0.443977 | ^4^ |
| 7 | 2007-2009 | Amazon, Ecuador | -0.6754 | -76.4429 | 0.29 | 0.178872 | ^4^ |
| 8 | 2003 | Quendeque, Madidi, Amazon, Bolivia | -14.9998 | -67.7931 | 0.27 | 0.260485 | ^5^ |
| 9 | 2004 | Upper Madidi, Amazon, Bolivia | -13.6005 | -68.7502 | 2.22 | 0.631798 | ^5^ |
| 10 | 2002-2008 | Tuichi-Hondo, Madidi, Amazon, Bolivia | -14.6001 | -67.6901 | 1.98 | 0.305409 | ^5^ |
| 11 | 2016-2017 | PERD, Atlantic Forest | -19.729326 | -42.554149 | 0.76 | 0.162563 | ^6^ |
| 12 | 2014-2015 | Jaguar Northern Reserve, Sahuaripa, Sonora | 29.46668152 | -109.1670653 | 1.07 | 0.014422 | ^7^ |
| 13 | 2009 | Nacori Chico village, Sonora | 29.68862979 | -108.9744721 | 1.54 | 0.003311 | ^8^ |
| 14 | 2014 | San José de Los Pinos, Michoacán | 18.17811663 | -102.516495 | 0.75 | 0.11358 | ^9^ |
| 15 | 2015 | Oaxaca | 18.01953282 | -96.53295899 | 1.16 | 0.012183 | ^10^ |
| 16 | 2019-2020 | Nayarit, Mangroves | 21.677971 | -105.439576 | 2.35 | 0.009562 | ^11^ |
| 17 | 2016 | Campeche | 18.330556 | -91.763889 | 1.93 | 0.666915 | ^12^ |
| 18 | 2013-2014 | Cockscomb Basin Wildlife Sanctuary, Belize | 16.89337146 | -88.63772815 | 2.01 | 0.248315 | ^13^ |
| 19 | 2011-2014 | Ranchs, Pantanal | -17.361279 | -56.72257 | 4.08 | 0.088838 | ^14^ |
| 20 | 2011-2014 | Near Serra do Amolar, Pantanal | -17.808839 | -57.582707 | 3.59 | 0.349034 | ^14^ |
| 22 | 2014-2018 | Taiamã Ecological Station, Pantanal | -16.898351 | -57.489603 | 12.4 | 0.303388 | ^15^ |
| 23 | 2010 | Emas National Park, Cerrado | -18.1127171 | -52.91046398 | 0.17 | 0.118653 | ^16^ |
| 24 | 2016-2017 | Santa Rosa, Guanacaste Conservation Area, Costa Rica | 10.826708 | -85.653633 | 7.6 | 0.583892 | ^17^ |
| 25 | 2016-2018 | Site 1, Dry Chaco, Paraguay | -20.707466 | -60.039135 | 1.26 | 0.217479 | ^18^ |
| 26 | 2016-2018 | Site 2, Dry Chaco, Paraguay | -19.647921 | -58.621898 | 1.6 | 0.132929 | ^18^ |
| 27 | 2016-2018 | Site 3, Dry Chaco, Paraguay | -21.75072 | -60.050121 | 0.58 | 0.209789 | ^18^ |
| 28 | 2016-2018 | Site 4, Dry Chaco, Paraguay | -20.06512 | -59.366222 | 0.44 | 0.183024 | ^18^ |
| 29 | 2013 | Moist forest, Guatemala | 17.240727 | -89.19708 | 1.52 | 0.408925 | ^19^ |
| 30 | 2020 | Alto Turiaçu IL, Amazon | -3.042 | -46.4157 | 2.3 | 0.195899 | ^20^ |
| 31 | 2019 | Arapaxi RESEX, Amazon | -8.842683 | -67.840581 | 1.61 | 0.257086 | ^20^ |
| 32 | 2019 | Cabo Orange NP, Amazon | 2.93657 | -51.19991 | 3.22 | 0.350902 | ^20^ |
| 33 | 2018 | Cazumba-Iracema RESEX, Amazon | -9.449309 | -68.907706 | 2 | 0.154032 | ^20^ |
| 34 | 2019 | Chico Mendes RESEX, Amazon | -10.288559 | -68.17321 | 2.92 | 0.075097 | ^20^ |
| 35 | 2018-2020 | Cuieiras REBIO, Amazon | -2.57401 | -60.30432 | 1.78 | 0.425174 | ^20^ |
| 36 | 2018-2019 | Cuyabeno WR, Amazon | -0.325916 | -75.785472 | 2.94 | 0.533674 | ^20^ |
| 37 | 2011-2012 | Deni & Kanamari do Xeruã IL, Amazon | -6.60918 | -68.23544 | 2.39 | 0.508857 | ^20^ |
| 38 | 2019 | ESEC Maracá, Amazon | 3.39513 | -61.54202 | 2.58 | 0.289638 | ^20^ |
| 39 | 2016-2017 | ESEC Terra do Meio, Amazon | -4.685691 | -53.5808 | 3.57 | 0.285474 | ^20^ |
| 40 | 2009-2014 | Espinoza FC, Amazon | -11.46 | -69.7 | 3.39 | 0.285284 | ^20^ |
| 41 | 2016-2019 | FLONA Jamari, Amazon | -9.346536 | -62.966493 | 2.02 | 0.187772 | ^20^ |
| 42 | 2017-2019 | Güeppi-Sekime NP, Amazon | -0.370944 | -75.214722 | 2.73 | 0.466715 | ^20^ |
| 43 | 2020 | Gurupi REBIO, Amazon | -3.656 | -46.809 | 2.82 | 0.171871 | ^20^ |
| 44 | 2016-2017 | Jaú NP, Amazon | -1.85749 | -61.69167 | 1.08 | 0.395466 | ^20^ |
| 45 | 2019 | Juruena NP, Amazon | -8.985254 | -58.577 | 3.26 | 0.374892 | ^20^ |
| 46 | 2006-2010 | Los Amigos CC, Amazon | -12.54 | -70.06 | 4.37 | 0.382849 | ^20^ |
| 47 | 2019 | Médio Purus RESEX, Amazon | -7.75237 | -65.649433 | 3.23 | 0.375538 | ^20^ |
| 48 | 2013-2014 | SDR Amanã, Amazon | -2.47485 | -64.62042 | 5.17 | 0.597833 | ^20^ |
| 49 | 2013-2014 | SDR Mamirauá, Amazon | -3.04553 | -64.86188 | 8.94 | 0.643137 | ^20^ |
| 50 | 2007 | Tambopata NR & Bahuaja Sonene NP, Amazon | -12.99 | -69.46 | 1.82 | 0.414076 | ^20^ |
| 51 | 2018-2019 | Umancia IL, Amazon | -0.314944 | -74.375805 | 2.02 | 0.385838 | ^20^ |
| 52 | 2022 | Site I, Magdalena River Colombia | 6.033333 | -71.4 | 3.8 | 0.03476 | ^21^ |
| 53 | 2014 | Manu National Park, Amazon, Peru | -11.856921 | -71.453699 | 2.5 | 0.528602 | ^22^ |
| 54 | 2009 | Copo N.P., Argentina | -25.98 | -61.9 | 0 | 0.102371 | Quiroga et al., 2014, as cited in ^23^ |
| 55 | 2006-2007 | PN Iguazú, Iguazú-San Jorge, Argentina | -25.64 | -54.33 | 1.2 | 0.299698 | Paviolo et al., 2008; Paviolo et al., 2016, as cited in ^23^ |
| 56 | 2008 | Iguazú-Urugua-í, Green Corridor I and II, Argentina | -25.63 | -54.21 | 0.89 | 0.263647 | Paviolo et al., 2016, as cited in ^23^ |
| 57 | 2008 | Aborigen Region, Argentina | -24.76 | -62.18 | 0 | 0.124446 | Quiroga et al., 2014, as cited in ^23^ |
| 58 | 2010 | El Canton, Argentina | -23.36 | -62.23 | 0 | 0.090012 | Quiroga et al., 2014, as cited in ^23^ |
| 59 | 2008-2013 | Hill Bank, Belize | 17.61 | -88.79 | 4.24 | 0.476481 | Kelly et al., 2014, as cited in ^23^ |
| 60 | 2013 | Gallon Jug, Belize | 17.64 | -89.11 | 1.8 | 0.334832 | Kelly et al., 2014, as cited in ^23^ |
| 61 | 2008-2013 | La Milpa, Belize | 17.73 | -89.05 | 2.3 | 0.398255 | Kelly et al., 2014, as cited in ^23^ |
| 62 | 2004 | Guanaco II, Kaa-Iya II, Bolivia, Bolivia | -20.07 | -62.44 | 1.54 | 0.16912 | Cuéllar et al., 2004; Noss et al., 2012, as cited in ^23^ |
| 63 | 2002-2003 | Cerro cortado II, Gran Chaco National Park, Bolivia | -19.54 | -61.32 | 1.18 | 0.232521 | Maffei et al., 2002; Maffei et al., 2003; Noss et al., 2012, as cited in ^23^ |
| 64 | 2006-2007 | Palmar-Ravelo II, Kaa-Iya, Bolivia | -19.42 | -60.53 | 0.58 | 0.203844 | Romero-Muñoz et al., 2006; Romero-Muñoz et al., 2007; Romero-Muñoz et al., 2008; Montaño and Noss, 2010; Noss et al., 2012, as cited in ^23^ |
| 65 | 2003 | Ravelo I, Gran Chaco National Park, Bolivia | -19.32 | -60.61 | 0.59 | 0.172061 | Cuéllar et al., 2003a; 2003b; Peña et al., 2004; Noss et al., 2012, as cited in ^23^ |
| 66 | 2002-2004 | Tucavaca I, Gran Chaco National Park, Bolivia | -18.52 | -60.82 | 0.99 | 0.139787 | Maffei et al., 2002; Maffei et al., 2004; Noss et al., 2012, as cited in ^23^ |
| 67 | 2010-2011 | Ilha do Cardoso, Brazil | -25.11 | -47.94 | 0 | 0.153746 | Paviolo et al., 2016, as cited in ^23^ |
| 68 | 2012 | Juréia-Itatins, Brazil | -24.38 | -47.06 | 0 | 0.044525 | Paviolo et al., 2016, as cited in ^23^ |
| 69 | 2011 | Intervales-Petar, Brazil | -24.22 | -48.29 | 0.66 | 0.131818 | Paviolo et al., 2016, as cited in ^23^ |
| 70 | 2013 | Santa Virginia, Brazil | -23.36 | -45.2 | 0 | 0.059966 | Paviolo et al., 2016, as cited in ^23^ |
| 71 | 2008-2009 | Ivinhema, Brazil | -23.08 | -53.66 | 1.66 | 0.122123 | Paviolo et al., 2016, as cited in ^23^ |
| 72 | 2008-2009 | Serra da Bocaina, Brazil | -22.96 | -44.67 | 0 | 0.23185 | Paviolo et al., 2016, as cited in ^23^ |
| 73 | 2003 | Moro do Diablo, Brazil | -22.61 | -52.2 | 2.39 | 0.088483 | Cullen Jr. 2006; Paviolo et al., 2016, as cited in ^23^ |
| 74 | 2010-2011 | Serra dos Órgãos I, II, III and IV., Brazil | -22.56 | -43.13 | 0 | 0.000003 | Paviolo et al., 2016, as cited in ^23^ |
| 75 | 2005-2006 | Vale NR I, II, III, IV and V, Brazil | -19.09 | -39.91 | 2.42 | 0.157267 | Paviolo et al., 2016, as cited in ^23^ |
| 76 | 2007 | Caatinga-Serra da Capivara, Brazil | -8.86 | -42.61 | 1.45 | 0.037463 | Silveira et al., 2010; Sollmann et al., 2013, as cited in ^23^ |
| 77 | 2014 | Magdalena river, Colombia | 7.47 | -73.8 | 3.04 | 0.016858 | Boron et al., 2016, as cited in ^23^ |
| 78 | 2009 | Laguna del Tigre, Sur Corredor Biológico, Portion of Consecion AFISAP, Guatemala | 17.3 | -90.35 | 4.52 | 0.374862 | Moreira et al., 2009; Tobler et al., 2013, as cited in ^23^ |
| 79 | 2012-2013 | Charabaru Conssesion, Guyana | 4.89 | -58.44 | 4.48 | 0.314056 | Paemelaere et al., 2013, as cited in ^23^ |
| 80 | 2008-2012 | Reserva Ecológica El Eden, Mexico | 21.35 | -87.35 | 1.95 | 0.628039 | Ávila-Nájera et al., 2015, as cited in ^23^ |
| 81 | 2013-2014 | Hato Piñero, Venezuela | 8.9 | -68.11 | 4.65 | 0.087615 | Jȩdrzejewski et al., 2017, as cited in ^23^ |

**Table S3. Assessment of** **the estimated amount of suitable habitat for jaguars (*Panthera onca*) distributed across the species current range, non-designated lands, jaguar conservation units (JCUs) and protected areas (PAs).** Total summed: the total summed habitat suitability (km²); Highly suitable area: corresponds to the area (km²) classified as highly suitable habitat (top quartile of suitability); Range-based: refers to the suitable habitat identified when the entire current range was analysed as a single unit; Ecoregion-based: reflects the suitable habitat identified when each ecoregion was analysed separately.

| **Region** | **Habitat Suitability** | | |
| --- | --- | --- | --- |
|  | **Total summed (%)** | **Highly suitable area** | |
|  |  | **Range-based (%)** | **Ecoregion-based (%)** |
| Current range | 8,766,692.9 (100) | 14,584,552 (100) | 14,578,260 (100) |
| Non-Designated Lands | 862,151.2 (9.8) | 1,673,350 (11.5) | 1,394,212 (9.7) |
| JCUs | 6,023,761.1 (68.7) | 11,190,394 (76.7) | 9,063,221 (62.2) |
| PAs | 4,721,829 (53.9) | 8,603,742 (59.0) | 6,868,576 (47.1) |


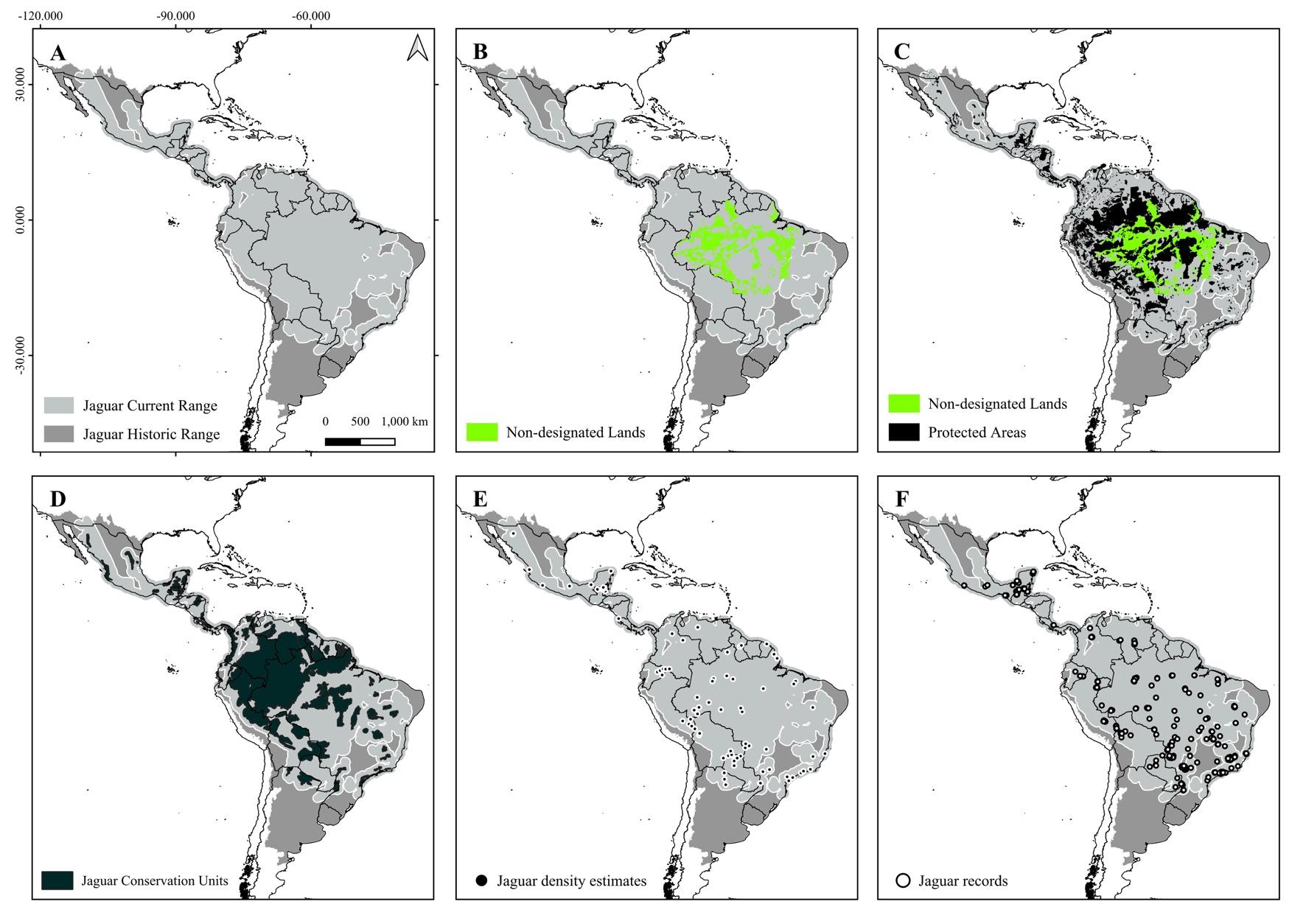


**Figure S1. Reference maps depicting jaguar historic and current range (A), non-designated lands (B), protected areas (Indigenous Lands included – C), Jaguar Conservation Units (D), jaguar density estimates used in models’ comparison (E), and jaguar records used for validation (F).** Figure created using QGIS v3.36.0 (https://qgis.org).


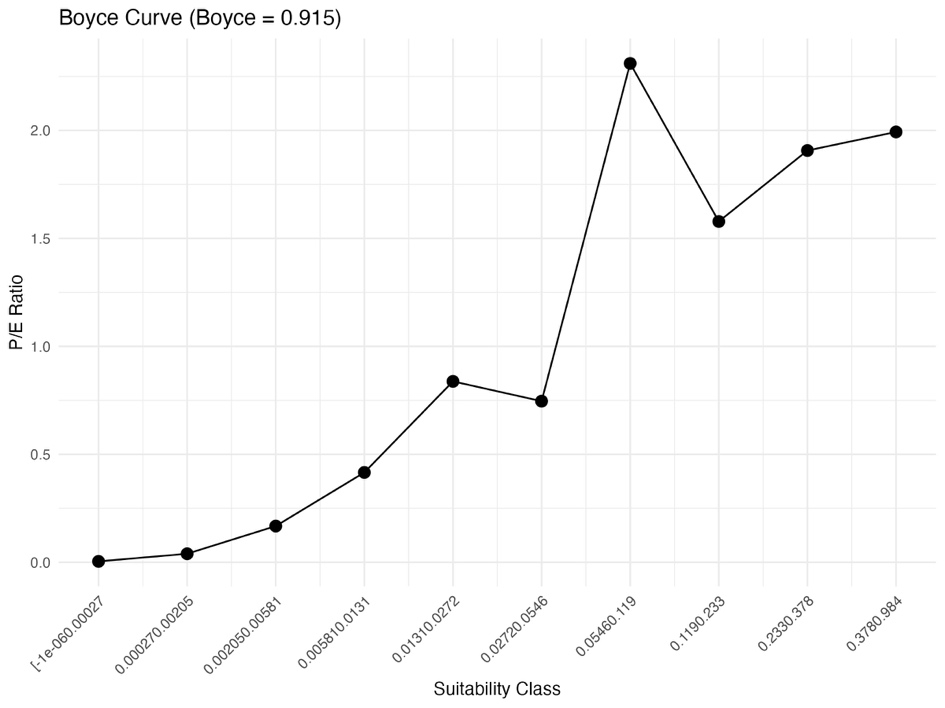


**Figure S2. Boyce Index Evaluation Curve.** The curve shows the Predicted-to-Expected (P/E) ratio across suitability classes of the model. Values above 1 indicate that presences occur more frequently than expected under random distribution, reflecting good model performance. The overall Boyce Index for the model is 0.91, indicating strong predictive power.


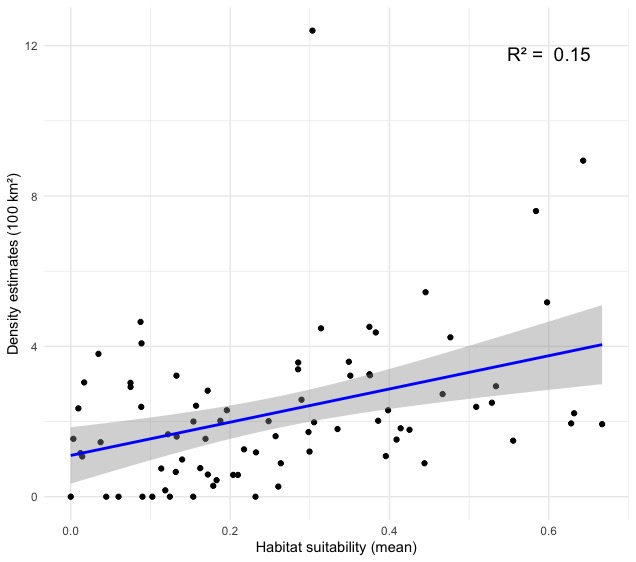


**Figure S3. Linear regression showing a positive relationship between jaguar density estimates and the habitat suitability model.**

**
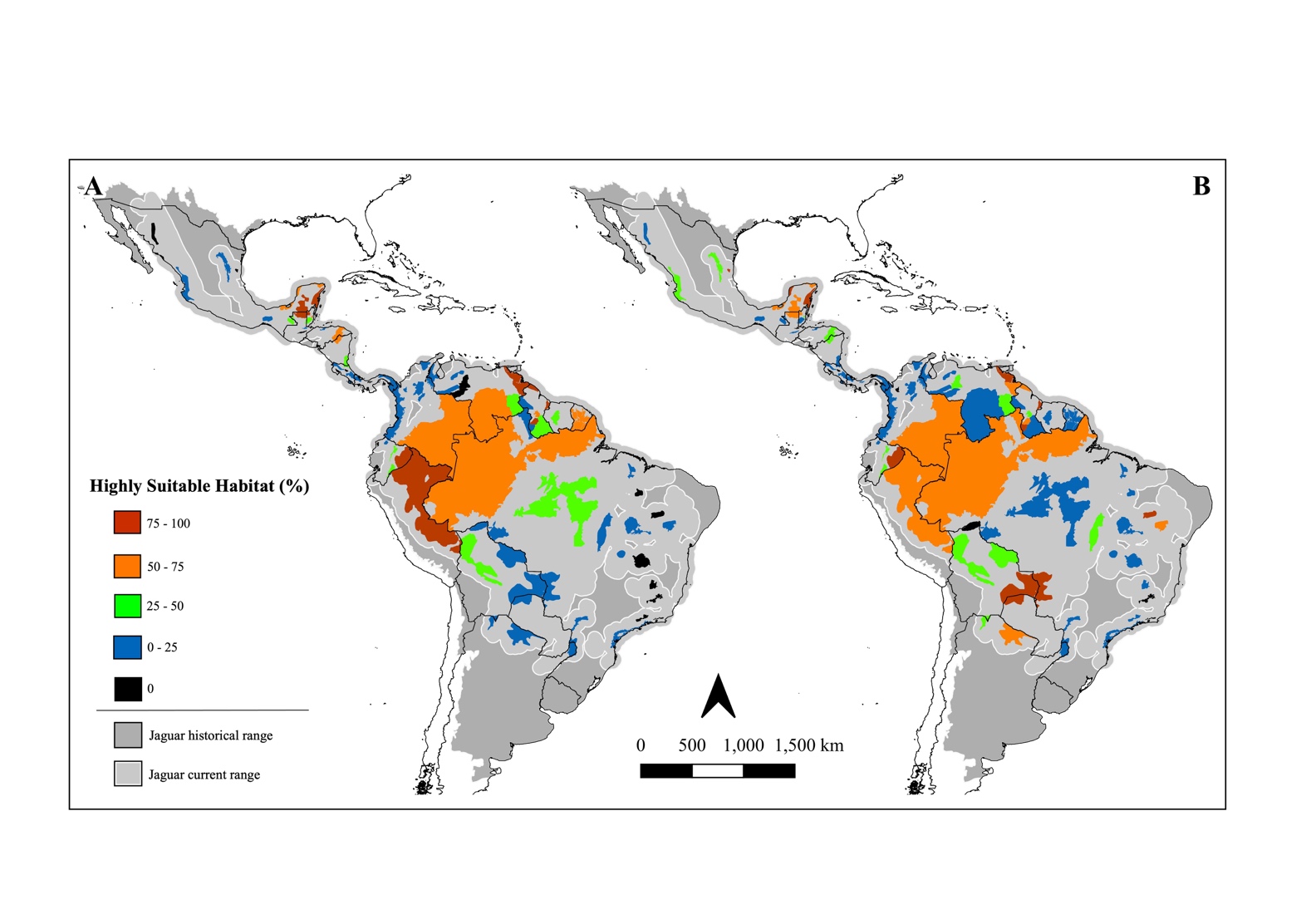
**

**Figure S4.** **The Jaguar Conservation Units (JCUs) classified by the percentage of highly suitable areas relative to their respective areas considering the range-based (A) and ecoregion-based scenarios (B).** Figure created using QGIS v3.36.0 (https://qgis.org).

**
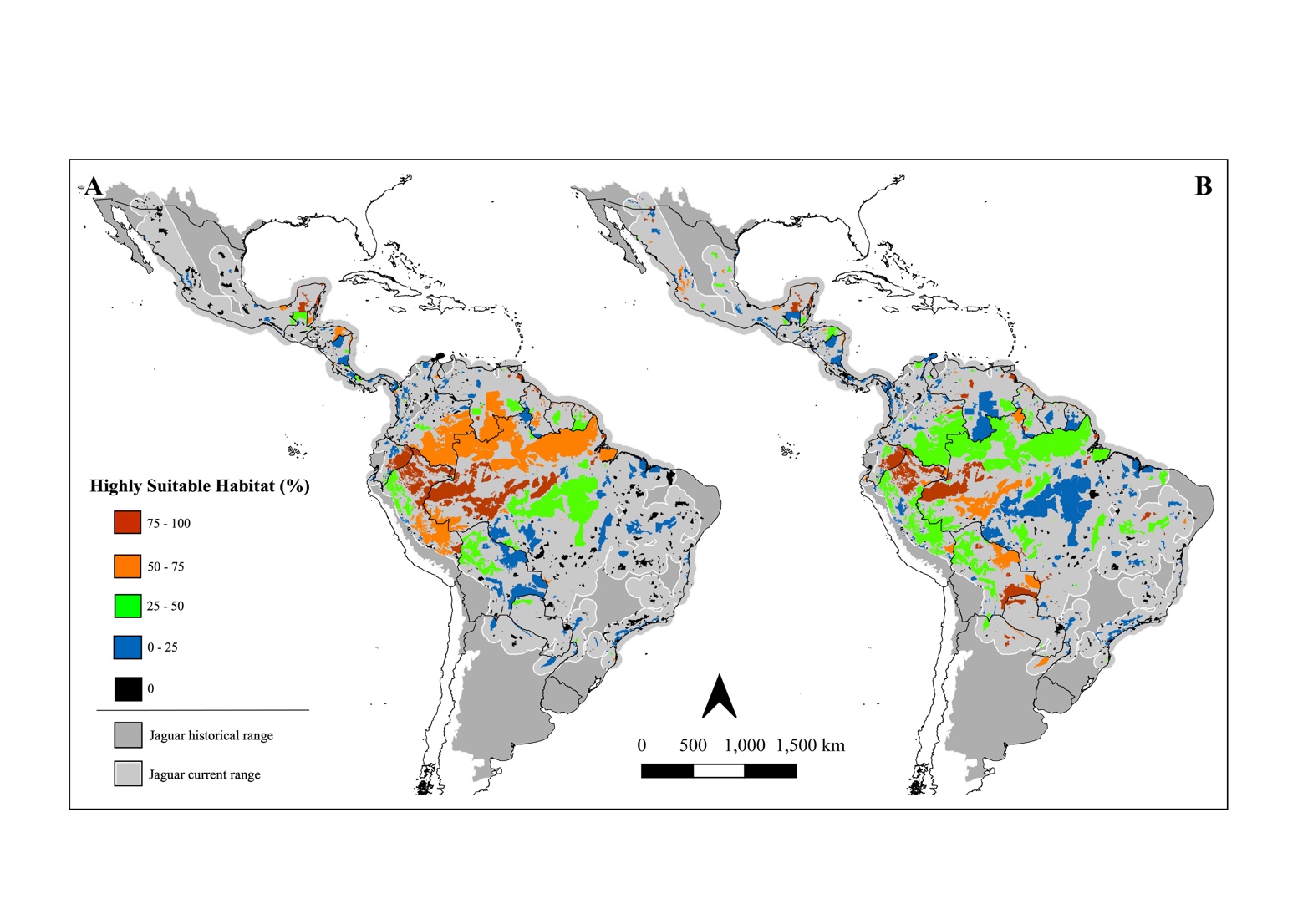
**

**Figure S5.** **The Protected Areas and Indigenous Lands PAs classified by the percentage of highly suitable areas relative to their respective areas considering the range-based (A) and ecoregion-based scenarios (B).** Figure created using QGIS v3.36.0 (https://qgis.org).

**References Supplementary**

1. Trinca, C. T. Densidade populacional de felídeos e riqueza de mamíferos terrestres no sul da Amazônia. Universidade Federal da Paraíba (UFPB), 2014.

2. Roopsind, A., Caughlin, T. T., Sambhu, H., Fragoso, J. M. V. & Putz, F. E. Logging and indigenous hunting impacts on persistence of large Neotropical animals. Biotropica 49, 565–575 (2017).

3. Petit, M., Denis, T., Rux, O., Richard-Hansen, C. & Berzins, R. Estimating jaguar (*Panthera onca*) density in a preserved coastal area of French Guiana. Mammalia 82, 188–192 (2018).

4. Espinosa, S., Celis, G. & Branch, L. C. When roads appear jaguars decline: Increased access to an Amazonian wilderness area reduces potential for jaguar conservation. PLoS One 13, 1–18 (2018).

5. Ayala, G., Viscarra, M. E., Fonseca, C. & Wallace, R. B. Estimates of jaguar (*Panthera onca*) population density in the South American Greater Madidi- Tambopata Landscape. Revista de Ciencias Ambientales (Tropical Journal of Environmental Sciences) 56, 1–16 (2022).

6. de Azevedo, F. C. C., Pasa, J. B., Arrais, R. C., Massara, R. L. & Widmer, C. E. Density and habitat use of one of the last jaguar populations of the Brazilian Atlantic Forest: Is there still hope? Ecol Evol 12, 1–15 (2022).

7. Amador-Alcalá, S. A. et al. Abundance of the northernmost jaguar *Panthera onca* breeding population. Anim Biodivers Conserv 47, 49–61 (2024).

8. Greenspan, E., Anile, S. & Nielsen, C. K. Density of wild felids in Sonora, Mexico: a comparison of spatially explicit capture-recapture methods. Eur J Wildl Res 66, 1–13 (2020).

9. Charre-Medellín, J. F., Monterrubio-Rico, T. C., Acevedo, P., Guzmán-Díaz, E. O. & Jiménez, J. Jaguar (*Panthera onca*) density in the Sierra Madre del Sur; the last wilderness area in the central-western slope in Mexico. Stud Neotrop Fauna Environ 58, 47–60 (2023).

10. Lavariega, M. C. et al. Community-Based Monitoring of Jaguar (*Panthera onca*) in the Chinantla Region, Mexico. Trop Conserv Sci 13, (2020).

11. Luja, V. H., Guzmán-Báez, D. J., Nájera, O. & Vega-Frutis, R. Jaguars in the matrix: population, prey abundance and land-cover change in a fragmented landscape in western Mexico. Oryx 56, 546–554 (2022).

12. Hidalgo-Mihart, M. G. et al. Jaguar density in a mosaic of disturbed/preserved areas in southeastern Mexico. Mammalian Biology 98, 173–178 (2019).

13. Harmsen, B. J., Foster, R. J. & Quigley, H. Spatially explicit capture recapture density estimates: Robustness, accuracy and precision in a long-term study of jaguars (*Panthera onca*). PLoS One 15, 1–19 (2020).

14. Devlin, A. L. et al. Drivers of large carnivore density in non-hunted, multi-use landscapes. Conserv Sci Pract 5, 1–13 (2023).

15. Eriksson, C. E. et al. Extensive aquatic subsidies lead to territorial breakdown and high density of an apex predator. Ecology e03543 (2021) doi:10.1002/ecy.3543.

16. Finnegan, S. P. et al. Reserve size, dispersal and population viability in wide ranging carnivores: the case of jaguars in Emas National Park, Brazil. Anim Conserv 24, 3–14 (2021).

17. Montalvo, V. H., Sáenz-Bolaños, C., Cruz-Díaz, J. C., Carrillo, E. & Fuller, T. K. The Use of Camera Traps and Auxiliary Satellite Telemetry to Estimate Jaguar Population Density in Northwestern Costa Rica. Animals 12, 2544 (2022).

18. Thompson, J. J. et al. Jaguar (*Panthera onca*) population density and landscape connectivity in a deforestation hotspot: The Paraguayan Dry Chaco as a case study. Perspect Ecol Conserv (2022) doi:10.1016/j.pecon.2022.09.001.

19. Tobler, M. W. et al. Do responsibly managed logging concessions adequately protect jaguars and other large and medium-sized mammals? Two case studies from Guatemala and Peru. Biol Conserv 220, 245–253 (2018).

20. Alvarenga, G. C. et al. Jaguar (*Panthera onca*) density and population size across protected areas and indigenous lands in the Amazon biome, its largest stronghold. Biol Conserv 303, 111010 (2025).

21. Hyde, M. et al. Tourism-supported working lands sustain a growing jaguar population in the Colombian Llanos. Sci Rep 13, 1–11 (2023).

22. Maffei, L. et al. Assessment of jaguars *Panthera onca* (Mammalia: Carnivora: Felidae) and their prey in Manu National Park. Mammalogy Notes 7, 267 (2021).

23. Jȩdrzejewski, W. et al. Estimating large carnivore populations at global scale based on spatial predictions of density and distribution - Application to the jaguar (*Panthera onca*). PLoS One 13, 1–25 (2018).
